# A framework for chemical safety assessment incorporating new approach methodologies within REACH

**DOI:** 10.1007/s00204-021-03215-9

**Published:** 2022-02-01

**Authors:** Nicholas Ball, Remi Bars, Philip A. Botham, Andreea Cuciureanu, Mark T. D. Cronin, John E. Doe, Tatsiana Dudzina, Timothy W. Gant, Marcel Leist, Bennard van Ravenzwaay

**Affiliations:** 1 Dow Europe, Toxicology and Environmental Research and Consulting, Bachtobelstrasse 3, 8810 Horgen, Switzerland; 2 Bayer CropScience, 355 rue Dostoïevski, 06903 Sophia Antipolis, France; 3 Syngenta Product Safety, Jealott’s Hill International Research Centre, Bracknell, RG42 6EY Berkshire UK; 4 ECETOC AISBL, Rue Belliard 40, 1040 Bruxelles, Belgium; 5 School of Pharmacy and Biomolecular Sciences, Liverpool John Moores University, Byrom Street, Liverpool, L3 3AF UK; 6 ExxonMobilBiomedical Sciences Inc, Hermeslaan 2, 1831 Machelen, Belgium; 7 NIHR Health Protection Research Unit in Environmental Exposures and Health, Imperial College London, London, UK; 8 CAAT-Europe and Department of Biology, University of Konstanz, 78464 Konstanz, Germany; 9 BASF SE, Experimental Toxicology and Ecology, Ludwigshafen, Germany

**Keywords:** Chemical risk assessment, Toxicity, New approach methodology, Tiered assessment, Regulatory framework

## Abstract

The long-term investment in new approach methodologies (NAMs) within the EU and other parts of the world is beginning to result in an emerging consensus of how to use information from in silico, in vitro and targeted in vivo sources to assess the safety of chemicals. However, this methodology is being adopted very slowly for regulatory purposes. Here, we have developed a framework incorporating in silico*, *in vitro and in vivo methods designed to meet the requirements of REACH in which both hazard and exposure can be assessed using a tiered approach. The outputs from each tier are classification categories, safe doses, and risk assessments, and progress through the tiers depends on the output from previous tiers. We have exemplified the use of the framework with three examples. The outputs were the same or more conservative than parallel assessments based on conventional studies. The framework allows a transparent and phased introduction of NAMs in chemical safety assessment and enables science-based safety decisions which provide the same level of public health protection using fewer animals, taking less time, and using less financial and expert resource. Furthermore, it would also allow new methods to be incorporated as they develop through continuous selective evolution rather than periodic revolution.

**Supplementary Information:**

The online version contains supplementary material available at 10.1007/s00204-021-03215-9.

## Background

Chemicals have many uses which benefit society but also have the potential to cause harm. For this reason, a regulatory system has evolved over the last 50 years to meet the aim of allowing the use of chemicals to benefit society without causing harm to human health and the environment. To date, this has relied largely on animal-based approaches which are expensive and rapidly becoming unacceptable to large parts of society. There are also concerns over the transferability of animal data to humans. The change in attitude has been paralleled by rapid advancements in in silico and in vitro methods built on the back of the developments in technology, computing, and molecular biology. This has led to a huge investment by government, industry, and academia to develop new approach methodology (NAMs) based on these advancements for the assessment of hazard and risk of chemicals. Technical progress has been made, but fitting new technology into the existing regulatory system is proving to be difficult for many reasons, scientific and otherwise. There have been many programmes heralding the need for reform fit for the 21st century including the US Federal Program Tox21 (Thomas et al. 2018), HESI’s programme Risk21 (Pastoor et al. 2014), and EU-ToxRisk21 (Moné et al. 2020), but a quarter of the twenty-first century will have soon passed, and the old system is still in place.

Much of the technology which is required to provide a twenty-first century system already exists, but a revised framework needs to be developed to use it to meet the goal of handling chemicals without risk. There must be good reasons to make changes and these can be summarised as: meeting the goal of protecting human health more rapidly, using less resources (fewer animals, quicker data collection), and using interpretation approaches that better facilitate regulatory review (Thomas et al. 2018).

We are now at a watershed moment to capitalise on prolonged investment in research into NAMs, with over €650 million since 2000 by the EU (Knight et al. 2021). There are no published figures for how much has been spent in USA, but it can be assumed to be, as a minimum, a similar amount. There is increased scientific knowledge on how chemicals cause adverse effects; new methodologies in vitro and in silico which can assess biological pathways; and progress in integrating the evidence from disparate sources. We must now come to a scientific consensus on how to put these factors together for chemical safety assessment into a tiered approach integrating existing knowledge, data on biological activity, and assessment of toxicokinetics from in vitro and in vivo sources (Mahoney et al. 2020). In addition, we have a decade of data development for chemicals under REACH and other regulations, a treasure which can, and should, be used in a new framework to provide validation of NAMs, in assessments such as read across, as well as for the development of in silico predictions in a digitalized toxicological universe (Rovida et al. 2020; Krebs et al. 2020; Patterson et al. 2021; Golden et al. 2020; Luechtefeld et al. 2018). However, it should be remembered that the goal is to assess safety for humans not to reproduce the results of laboratory animal studies (van Norman 2019).

To date, there has been a focus on study-by-study or method-by-method replacements of animal tests with NAMs, but overall this approach has had only limited success. This approach has been mainly successful for simple acute endpoints/hazards (such as skin and eye irritation). For complex hazards, such as reproductive toxicity requiring a whole organism approach, this strategy is unlikely to be successful. The outcome of a hazard assessment process should be judged on whether an adequate hazard profile results in terms of type and category of hazard and setting of safe dose limits, not on whether all the endpoints from individual conventional toxicology studies could be predicted. It has been argued that the acceptability of a new approach to chemical safety assessment should be judged on whether it provides results which allow appropriate risk management actions to protect human health (Daston et al. 2015; Herzler et al. 2021; Lanzoni et al. 2019; Moné et al. 2020; Tralau et al. 2015).

Our aim here was to investigate whether a framework could be produced in which science-based safety decisions could be made that provide adequate levels of safety using fewer animals, taking less time, and using less financial and expert resource but operating as far as possible within the existing regulatory guidelines. We chose the current REACH regulatory guidelines to be the setting for our investigation, but the results are applicable to other regulatory systems.

## Framework and philosophy

The aim of a human health chemical safety assessment is to determine whether or not the use or presence of a chemical in the environment resulting in human exposure is reasonably certain to cause no harm to health. This requires knowledge of both the potential harm and the situation being assessed in terms of human exposure culminating in a risk assessment.

In addition to risk assessment, classification of identified hazards is part of the process. As stated in the EU CLP Guidance (ECHA 2017): “Hazard classification is a process involving identification of the physical, health and environmental hazards of a chemical or a mixture, followed by comparison of those hazards (including degree of hazard) with defined criteria in order to arrive at a classification of the chemical or mixture”. If the type of hazard is identified and the degree of hazard is known, then sensible judgements can be made regarding the use of the chemical. Degree of hazard is based on analysis of severity and potency. The current classification and labelling system has eight divisions of health hazard: acute toxicity, skin corrosion/irritation, eye damage/irritation, respiratory/skin sensitisation, germ cell mutagenicity, carcinogenicity, reproductive toxicity (includes developmental toxicity—DART), and specific target organ toxicity (STOT).

The chemical safety assessment process also requires a set of safe limit doses such as derived no/minimal effect levels (DNELs), acceptable daily intakes (ADIs), and acceptable operator exposure levels (AOELs), which estimate the dose which is safe for human exposure in different scenarios based on duration and route of exposure.

The potential harm can be quantified with different levels of certainty as continuous or discontinuous variables. Safe limits such as DNELs, AOELs, and ADIs are continuous variables for estimation of a safe dose for the hazard. Classification by hazard into bands or categories produces discontinuous variables for hazard.

Classification of hazard into bands or categories provides an estimate of hazard which can be used in a variety of situations (Doe et al. 2021). Examples include labelling of products with risk phrases, guidance on precautions to be used for transportation, and providing generic guidance on the suitability of products for general use scenarios—for instance, plant protection products may not be authorised for use by non-professional users if they are classified as acute toxicity category 1 and 2 or systemic target organ toxicity—single exposure (STOT-SE) category 1. Such generic guidance rarely takes into consideration whether exposures exceed reference doses for the uses in question and typically assumes that safety cannot be concluded at any exposure level (Generic Approach to Risk Management—Chemicals Strategy for Sustainability, EU 2020).

There are recommended sets of studies and guidance on how to interpret them for each hazard classification and for the derivation of reference values. These rely heavily on laboratory animal studies, but alternative methods have been incorporated into the process for some hazards.

Most chemicals currently in use do not have the full range of assays carried out on them and it is therefore not possible to develop a complete category and safe dose profile for them. Existing chemicals are considered on a case-by-case basis taking into account the overall tonnage produced and what is known about the chemical, which results in a wide variety of completeness of the category and safe dose profile. Chemicals must have a list of assays performed depending on the annual tonnage produced or imported, the larger the tonnage the larger the number of studies.

On the surface, it appears like a difficult task to generate a sufficiently complete category and safe dose profile for all chemicals. A pivotal question is whether eight health hazards may be covered using fewer resources per chemical.

## Assignment to hazard category and derivation of DNELs/AOEL/ADIs using a tiered approach

There are some potential inroads into addressing this problem arising from increased knowledge of the toxicology of chemicals and their use. The current methodology is based on what might be called “observational trials” rather than scientific investigations. The guideline studies seek to reproduce exaggerated human exposure situations by dosing animals with chemicals to see what happens. For example, chemicals are placed on the skin and the skin observed to see if damage results. Chemicals are applied and then dosed again some time later to see if sensitisation occurs. Animals are dosed to see if they survive a single exposure. Animals are dosed for 3 months to see what could happen after repeated dose. Animals are dosed for nearly all their lifetimes to see if they develop cancer. Animals are dosed during pregnancy and development to see if reproduction and development are affected. Many of these studies use doses which are much greater than is applicable to mimic human exposure. This brings a level of conservatism to the approach, although there are concerns over the relevance of doses which cause excessive toxicity or saturate metabolism pathways (ECETOC 2021a).

A vast body of knowledge has been accumulated over the near century that these “observational trials” have been in use. It has been realised that the observed “adverse outcomes” are caused by the effects of chemicals on biological processes and that the adverse outcomes can be predicted by knowing which processes or pathways are being affected (Leist et al. 2017). This concept has been used initially in the screening of chemicals in product selection in industries, for instance pharmaceutical and agrochemical, so that only acceptable chemicals are tested in the full list of mandated regulatory studies. Over a period of time, experience has shown that it is possible to choose chemicals that are unlikely to have an adverse categorisation profile when the full range of studies is eventually performed during the development of the chemical. These methods look at precursors of the adverse outcomes seen in the longer term trials using in silico comparison based on chemical structure, in vitro assessment in assays which model a range of pathways, and targeted in vivo assays. The OECD (2021a) is running a project which is systematically collecting case studies to evaluate this concept. In this paper, we have evaluated three applications of this approach.

Tiered approaches are based on the assumption that adverse outcomes can be predicted with greater precision, in terms of the presence or absence of a hazard and the degree of hazard including DNEL, as the process proceeds through in silico, to in vitro to in vivo (Leist et al. 2014). Thus, at each stage, the probability of a hazard and its degree and the resulting category and safe dose profile can be developed with increasing certainty. An initial problem formulation determines the level of precision which is required to make appropriate safety assessments at each tier.

It was noted earlier that most chemicals do not have a full set of studies which allow classification and categorisation in all eight health hazards and safe doses for all situations, yet they find a variety of uses. One reason for this is that for some uses it is not necessary to have a complete profile; for instance, chemical intermediates which are never isolated are not considered to need a full package, because human exposure in terms of number of people and dose will be very limited. It was also noted that the list of studies required for a new general chemical depends on the annual tonnage—this is used as a surrogate for the number of people exposed and dose. This implies that the different parts of the category and safe dose profile are required with different levels of certainty in different situations that can be characterised by different exposures. For instance, in the example quoted previously for plant protection products, the use by consumers of a category 1 chemical would not be allowed, and if there was a high level of certainty that the chemical was not category 1, then it may not be necessary to distinguish between category 2, 3, and not classified. Similarly, if there was a high degree of certainty that a chemical did not possess the hazard at all, that would be sufficient.

To utilise the outputs from a tiered approach to hazard identification and characterisation with the concept of a category and safe dose profile with different levels of certainty, there would need to be a tiered approach for each of the 8 hazard areas in CLP. We have exemplified this approach for repeat dose toxicity in “Examples of the tiered approach hazard and exposure framework”. Decisions on the safety of the use of chemicals are driven by the category within each hazard and the relevant safe dose such as a DNEL. The output of the process should be to assign a category and a DNEL with a level of confidence which allows decisions to be made.

The process could stop when an acceptable profile was reached, or it was determined that no acceptable profile would result. The acceptability of the category and safe dose profile would depend on the situation. Each situation would have a minimum acceptable category and safe dose profile associated with it based on the exposure situations arising from the situation. It would be necessary to go through the process again if new uses were to be investigated.

**Fig. 1:**
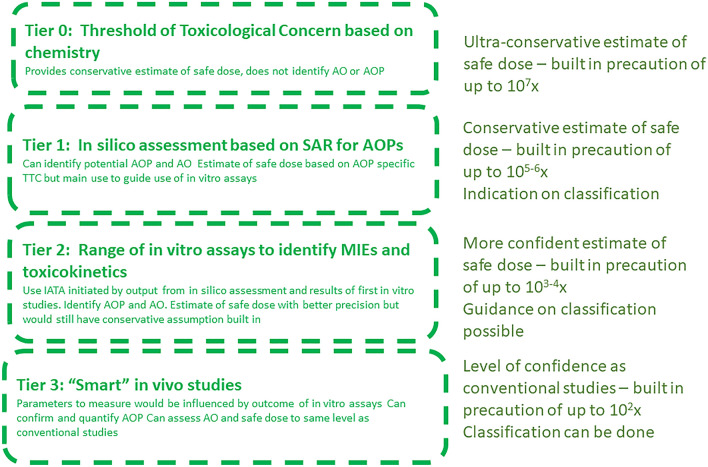
Generalised tiered approach to hazard assessment

This type of process has been developed in the concept of the Integrated Approach to Testing and Assessment (IATA) (OECD 2016, 2021a), where information from in silico, in vitro, and in vivo sources is considered within a structured framework. In some cases, the IATA uses cross-referencing to other compounds in a read-across procedure (van der Stel et al. 2021), in others, a Bayesian statistical approach to integrate the information coming from the various sources, for instance in assessing dermal sensitisation (Jaworska et al. 2013) or acute lethality (Firman et al. 2022). This approach could be extended to other hazards. Figure 1 shows a generalised framework for a tiered approach to hazard assessment. This is not a screening process, because information is generated at each tier which can be used for safety assessment in the appropriate situation. Assays are not evaluated for their ability to replace existing assays, but they are placed within a framework designed to generate the probability of which hazard category is appropriate for the chemical. Figure 1 provides an estimate of the level of precaution based on uncertainty at each tier. At Tier 0, based on the TTC, the assumption is made that all chemicals in the same class have the potency of the most potent member of the class, although the range of potency within each class covers up to seven orders of magnitude (Tluczkiewicz et al. 2011). Thus, a chemical could be up to 10^7^ times less potent than it is assumed to be. The results of conventional animal studies which could be in Tier 3 are adjusted by factors of 2 or 3 orders of magnitude to derive a DNEL to allow for the possibility of inter- and intra-species variability. It is assumed that all chemicals show this level of variability, even though this may not be the case. Friedman et al. (2019) compared the results of Tier 2 in vitro assessments with Tier 3 in vivo assessments based on conventional studies. They showed that the median distributions of the estimates of the in vitro points of departure were 1–2 orders of magnitude lower than for the in vivo estimates. Thus, Tier 2 could be considered to have 1–2 additional orders of magnitude of uncertainty over Tier 3. Comparable indicators are not available for Tier 1 in silico assessments, but it is not unreasonable to suggest that Tier 1 could lie between Tier 0 and Tier 1. That would then give estimates of precaution for each tier, as shown in Fig. 1.

The major change in approach which is required to extend the concept lies in the problem formulation which precedes the assessment. Currently, the first question to be asked in most situations is “what is the list of studies mandated by the annual tonnage?” The studies are then performed, and the hazard category and/or DNEL is determined. Then, the next question becomes “is this chemical suitable for the situation of concern?”.

When assessing the risk for a chemical, a revised problem formulation would first ask “what is an acceptable hazard/DNEL profile for this situation?” This could be expressed in two ways; saying what the hazard/DNEL profile should be or what the hazard profile/DNEL should not be. To answer both, one would first need to know an (realistic) exposure level estimated using credible tools for the situation of interest. Once this had been determined, the next question would be “what information do we need to determine if the hazard/DNEL profile is acceptable?” which would be answered by running the process until a decision can be made. This could be achieved by considering information from lower tiers or it may require information from higher tiers.

## Categorisation of exposure

There has long been an apparent contradiction between a hazard-based approach and a risk-based approach to chemical safety assessment which stems from how and when to include exposure in the process. The hazard-based approach argues that the nature of the hazard will determine the use of a chemical and the hazard must be identified and characterised first. The risk-based approach argues that the exposure determines the risk as much as the hazard, if the exposure is known then hazards can be assessed in a targeted way and safety decisions made. This can make hazard classification challenging as the study requirements for classification may not have been met. However, the hazard led approach can cause studies to be carried out unnecessarily that take time, use animals, and incur costs to registrants and regulators. For instance, a chemical used for short periods of time is unlikely to require long-term studies, or where the exposure route does not occur in relation to the hazard assessed. Conversely, a risk-based approach can lead to hazard data gaps when new uses for a chemical are considered, for instance when a chemical used for short periods of time is considered for longer term use which might entail a new assessment being made when the use or exposure route changes.

The current approach in REACH seems at first sight to be a hazard-based approach and mandates lists of studies to be carried out which address potential hazards. The extent of the mandated list is decided by the annual production tonnage of the chemical in question. In reality, the annual tonnage acts as a surrogate for the exposure, in terms of both dose and number of people exposed, using the assumption that the larger the tonnage, the larger the exposure. Therefore, in fact, this emulates a risk-based approach, but the chosen parameter (annual tonnage) is not a good estimate of exposure.

The drawback with this approach is that it can lead to a data package which is not appropriate for the proposed use; the package can either contain redundant studies or miss relevant areas. If relevant data are missing, it can be difficult to reach an agreement about how the data gap should be filled as this becomes both a scientific and regulatory issue. At best, there is a delay as new data are generated, or at worst, inappropriate surrogate data from other sources are used.

Eventually, agreements are made on the hazard data which are required based on the proposed use, and in effect, the process switches to being exposure-driven; the exposure dictates which additional data are required over and above the original tonnage-based mandated list. However, the process has to be argued on a case-by-case basis involving time delays and use of expert resources by both regulators and registrants.

Therefore, the debate about hazard-based or exposure-based approaches boils down to whether there is a better way to initiate the process which would avoid redundant data and/or data gaps and whether the process of generating data as exposure needs are explored could be better structured to provide more certainty for registrants and regulators.

We feel that these improvements are possible. There needs to be an agreed starting process which would be based on a broad description of the exposure and a suite of hazard information requirements based on the known exposure. Once this initial assessment had been made, the process would proceed using a tiered approach for both hazard and exposure.

**Fig. 2:**
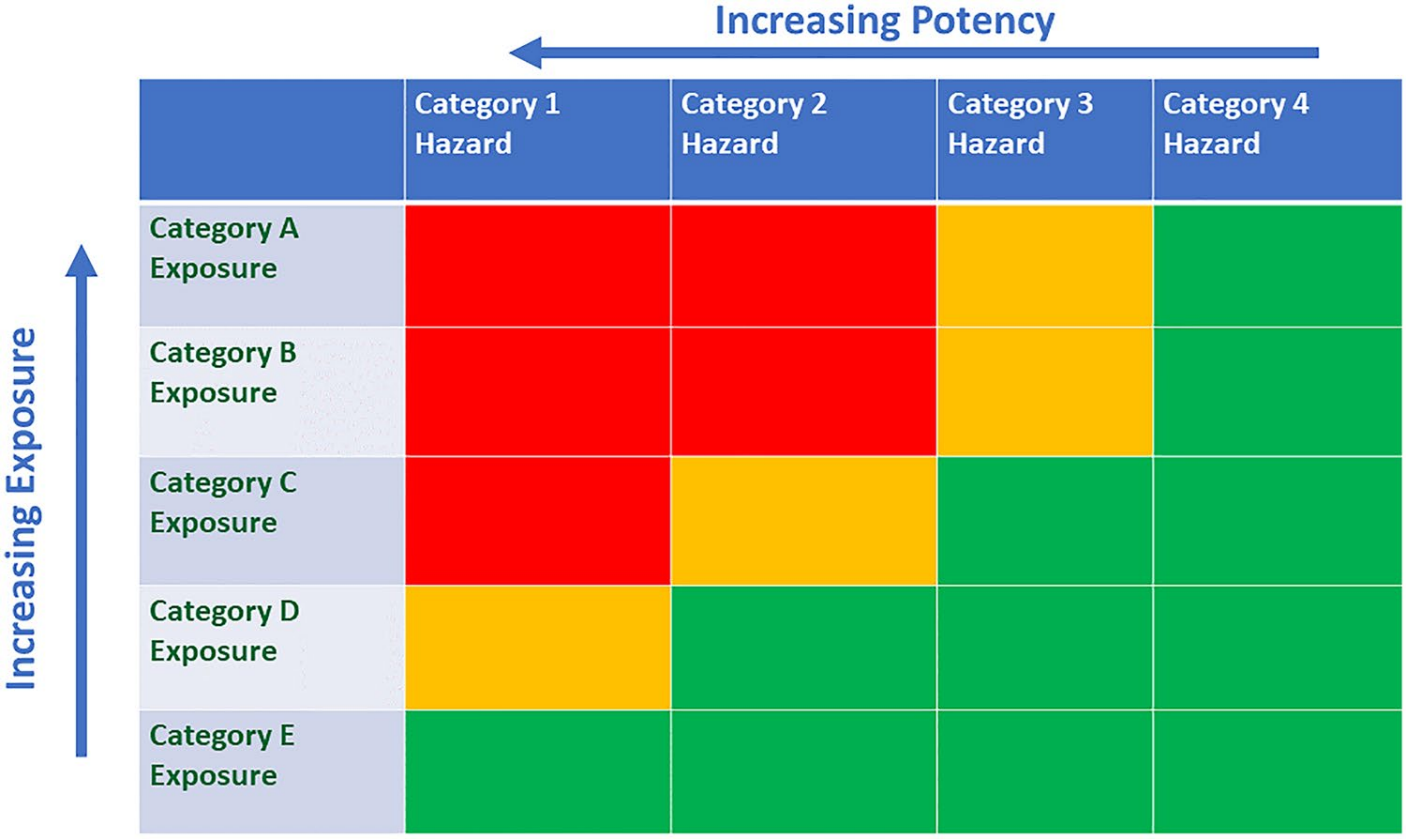
Schematic matrix of exposure and hazard categories. Green sectors indicate adequate margin of exposure; amber sectors indicate borderline margin of exposure; Red sectors indicate inadequate margin of exposure (color figure online)

Within CLP, hazards are categorised by degree of hazard, which is intended to be a simple way of informing the user/public about the hazard and to allow safety-based decisions on use and handling. If exposures were to be categorised in a similar way, then comparing hazard and exposure categories could be a simple aid to decision-making and communication. The matrix in Fig. 2 is a schematic for how this could work. Chemicals with a high degree of hazard would only be considered suitable in low exposure categories. Only chemicals with low degree of hazard would be considered suitable in high exposure categories.

## Exposure dose/duration categorisation

The starting point description of the exposure needs to consider both the level and the duration of exposure. The duration of exposure could be assigned into three periods: single- or 1-day exposure, repeat dose exposure up to 3 months, and longer term exposure. The dose could be assigned to one of five categories, with category A being the highest doses and category E being the lowest.

As far as possible, the exposure categories should be set to relate to the dose levels used to assign hazard categories within existing processes such as Classification Labelling and Packaging (CLP) and could also utilise the concept of the Threshold of Toxicological Concern (TTC). The values derived from CLP are shown in Table 1. This would enable the cross-referencing of exposure and hazard categories. (Note: The values for STOT-SE and STOT-RE Categories 3 and 4 are extrapolated from the dose ranges set in CLP for categories 1 and 2 to allow allocation of chemicals to one of four categories based on potency; the same principles about nature of the toxic effect as used to define category 1 or 2 are applied to these additional categories.)

**Table 1:** Dose level limits derived from CPL used in setting exposure categories

	Cat 1	Cat 2	Cat 3	Cat 4	Cat 5
Acute Oral (ATE)	< 5	5–50	50–300	300–2000	> 2000
STOT-SE (NOAEL)	< 300	300–2000	2000–5000	> 5000	
Dermal Sens (Conc)	< 0.1%	0.1–10%	10–100%	No response	
STOT-RE (NOAEL)	< 10	10–100	100–1000	> 1000	
Carcinogenicity SCL (T25)	< 1	1–100	> 100	No response	
Repro SCL (ED10)	< 4	4–400	> 400	No response	

The values are the dose levels from laboratory animal studies in mg/kg/day

Table 1 shows the dose-level limits which are based on doses used to assign chemicals to degree of hazard categories within CPL. The values are the dose levels from laboratory animal studies in mg/kg/day.

***Category E:*** The upper boundary of the lowest exposure category would be based on the Threshold of Toxicological Concern (TTC) (EFSA 2019)**,** a principle that refers to the establishment of a generic exposure level for all chemicals below which there would be a high probability of no risk to human health. Any substance is very unlikely to be potent enough to cause adverse effects at these dose levels. Therefore, even substances with category A Hazard could be used in scenarios associated with these low levels. The TTC has a range of values based on the chemistry of the substance being considered. The values which have been derived from animal data and are based on a 60 kg individual range from 0.0025 μg/kg/day for those with a structural alert for genotoxicity to 30 μg/kg/day for those in Cramer class I and already include uncertainty factor adjustment:

TTC values:

- With structural alert for genotoxicity: 0.0025 μg/kg/day.
- OPs and carbamates: 0.3 μg/kg/day.
- Cramer Class III: 1.5 μg/kg/day.
- Cramer Class II: 9.0 μg/kg/day.
- Cramer Class I: 30 μg/kg/day.

The TTC is based on long-term exposure and so they are appropriate for repeat exposure over 3 months. The structure should be checked to make sure it is within the applicability domain of the TTC and then the appropriate TTC should be used for the substance being considered; this does not require any experimentation. There is no overall basis to modify the TTC for shorter exposure durations apart from an overall understanding that the TTC is likely to be conservative for shorter durations, but the level of conservatism cannot be quantified. On the principle of being health protective, the appropriate TTC for the substance should be used even for short-term exposure.

***Category A:*** The highest exposure category boundary would be set by consideration of limit doses from toxicity studies. Category A exposures would only be considered safe for chemicals which showed no relevant toxicity at or above the limit dose in animal studies; these are categorised as Category 4. This is a dose which is considered to be the upper limit for dosing based on practical and ethical reasons. Effects produced above this level are not considered relevant.

Single dose lethality study limit dose: 2000 mg/kg.

Repeat dose limit dose: 1000 mg/kg.

It is health protective to set the boundaries for exposure to be low when there is uncertainty, so the widely used uncertainty factor of 100 (10 for interspecies and 10 for intra-species) could be used to derive lower boundaries for Category A exposures of:

One-day lower limit: 50 mg/kg.

Repeat dose up to 3 months lower limit: 10 mg/kg.

Repeat dose over 3 months lower limit: 10 mg/kg.

***Categories B–D***

Categories B–D would fill the range between the upper limit for Category E and the lower limit for Category A. The boundaries for categories B–D can be set by reference to the current boundaries for the degrees of hazard within the CLP system. The hazards of relevance are related to adverse effects caused by single or repeat dosing on general toxicity (STOT-SE and STOT-RE), carcinogenicity, and reproductive toxicity. The boundaries for the degree of hazard are different depending on the hazard and this raises the question of whether there should be one set of boundaries or whether the boundaries should depend on the hazard being considered. As the exposure categories could be considered as part of the process of decision-making on which hazard data should be generated, then there should be one set of boundaries for each duration of exposure period.

Using the principle of using lower limits to be health protective, then the lowest relevant limit between categories with an uncertainty factor applied should be used. The STOT hazard categories are based on the NOAEL from the relevant study. The hazard categories for carcinogenicity and for reproductive toxicity are based on the strength of evidence for the presence of the hazard rather than on degree of hazard and therefore pose a problem. However, the degree of hazard is taken into account when assessing specific concentration limits (SCL) in preparations for substances which have been classified for carcinogenicity and reproductive toxicity. They are divided into high, medium, and low potency bands based on results from the relevant studies—t25 for carcinogenicity and ED10 for reproductive toxicity (Doe et al 2021).

***One-day categorisation limits***—The most appropriate hazard for one day is STOT-SE. The limits for STOT-SE are

- Category 1: < 300 mg/kg.
- Category 2 300–2000 mg/kg.
- Category 3 2000–5000 mg/kg.
- Category 4 > 5000 mg/kg.

Applying the logic of using these values derived from animal studies and adjusting by an uncertainty factor of 100 to designate the boundaries, the following boundaries would be suggested:

- Category B 20–50 mg/kg.
- Category C 3–20 mg/kg.
- Category D specific TTC value—3 mg/kg.

***Repeat dose up to 3 month categorisation limits***

The most appropriate hazards for repeat dose up to 3 months would be STOT-RE and reproductive toxicity.

**Table:** 

	STOT-RE (NOAEL)	Repro (ED10)
Category 1	< 10	< 4
Category 2	10–100	4–400
Category 3	100–1000	> 400
Category 4	> 1000	No response

Using the principle of taking the health protective lower value and applying an uncertainty factor of 100, the following boundaries would be derived:

- Category B 4–10 mg/kg.
- Category C 40 μg/kg–4 mg/kg.
- Category D chemical specific TTC–40 μg/kg.

***Repeat dose over 3 month categorisation limits***

The most appropriate hazards for repeat dose exposure over 3 months would be STOT-RE, carcinogenicity, and reproductive toxicity.

**Table:** 

	STOT-RE	Carc (T25)	Repro (ED10)
Category 1	< 10	< 1	< 4
Category 2	10–100	1–100	4–400
Category 3	100–1000	> 100	> 400
Category 4	> 1000	No response	No response

Using the principle of taking the health protective lower value and applying an uncertainty factor of 100, the following boundaries would be derived from carcinogenicity which has the lowest indicative values:

- Category B 1–10 mg/kg.
- Category C 10 μg/kg–1 mg/kg.
- Category D Chemical specific TTC—10 μg/kg.

The route of exposure also needs to be considered, with appropriate adjustments made for dermal and inhalation. The utility of the concept depends upon the chemical’s use and the resulting exposure being known. The examples we have evaluated (“Examples of the tiered approach hazard and exposure framework”) show how this can be done and integrated into the assessment.

## Initiation of hazard assessment

The exposure dose/duration categories would be the starting point for the hazard evaluation. This would consist of a systematic review of each exposure dose/duration category for each of the hazard areas (acute lethality, eye and skin irritation, sensitisation, mutagenicity, STOT-SE and RE, carcinogenicity, and reproductive toxicity). The review would determine if additional information is required for the hazard area, mainly based on anticipated exposure duration. In addition, an estimate should be made of the minimum severity category and/or DNEL which would be acceptable for the use fitting into one of the exposure categories.

Once this has been done, the chemical would then enter the tiered approach for each relevant hazard category. If this strategy is consequently pursued, it is likely that a series of minimum information sets would emerge for each exposure dose/duration category. These would be analogous to the tonnage related minimum data set, but would be tailored for each usage. Table 2 contains the derived exposure category limits for each of the exposure periods.

**Table 2:** Exposure category limits. *Category E is based on the TTC and would vary depending on the genotoxicity and Cramer class of the chemical

	Cat A (mg/kg)	Cat B (mg/kg)	Cat C	Cat D	Cat E* (μg/kg)
1 day	> 50	20–50	3–20 mg/kg	0.0025 μg/kg–3 mg/kg	< 0.0025
Intermittent/short term	> 10	4–10	40 μg/kg–4 mg/kg	0.0025–40 μg/kg	< 0.0025
Long term	> 10	1–10	10 μg/kg–1 mg/kg	0.0025–10 μg/kg	< 0.0025

## Components of tiered approaches for hazard and exposure

NAMs are “new approaches”, not only based on their technological basis (non-animal test systems), but also because of the new concept they introduce to safety evaluation. Standard (animal-based) toxicological approaches often have (1) a stand-alone character and (2) provide direct data for regulatory decisions. This means that they do not require vast amounts of prior knowledge/accessory information, and they also usually do not necessitate complex interpretations in the light of other data. For instance, if the liver shows necrosis or severe hypertrophy in a 90-day study, this finding can be used directly to derive non-safe dose ranges and corresponding exposure limits.

Some of the older NAMs follow a similar concept. They allow, for instance, hazard assessment for phototoxicity, genotoxicity, or eye irritation by directly capturing apical phenotypic endpoints. However, the large majority of NAMs do not work this way (Leist et al. 2012a, b; Basketter et al. 2012), and their use follows different guiding principles: (1) the essential information is generally derived from a combination of several NAMs (not “stand-alone”); and (2) the outcome of NAMs is interpreted on the basis of a usually vast background knowledge, and after complex data processing. For instance, test data on the potency of a compound as an estrogen receptor agonist need to be combined with other experimental data on estrogen signalling and toxicokinetic behaviour, and physiological factors. All these data then feed complex algorithms that integrate extensive physiological and toxicological background knowledge to yield finally a threshold dose (Friedman et al. 2016, 2019; Casey et al. 2018; Beames et al. 2020) that may be used for regulatory decisions and risk management. This new approach can only work in practice if regulators have confidence in all the underlying processes and if they become familiar with using integrated approaches to testing and assessment (IATA) (OECD 2021a) as regulatory tools (“Box 1”).

Two fundamentally different applications of NAMs can be envisaged. They might be termed “data gap filling” and “ab initio assessment”. The former is already used, and it is well suited for a transition period. Typical applications are to complement animal studies by elucidating the mode of action, by identifying human-relevant metabolites or by predicting the bioactivity of minor metabolites and contaminants. In the future, NAMs may be used as a sole approach to assess unknown compounds. In this case, a large NAMs panel would be used simultaneously or consecutively, and even more NAMs may be added in higher tiers of testing. Such a NAMs battery would involve, e.g*., *in silico predictions of toxicokinetic properties and of likely biological targets (Leist et al. 2008, 2012a; Lanzoni et al. 2019; Friedman et al. 2019; Vinken et al. 2021). This information would be combined with in vitro tests that yield information on a chemical’s interaction with many defined biological structures and processes. In addition, data would be generated in complex test systems that yield information on the disturbance of any of thousands of genes or metabolites or other endpoints of untargeted omics technologies, which, it could be argued, cover a wider range of potential biological effects than covered by current apical effect-based animal studies.

The use of NAMs for complex toxicological endpoints (DART, STOT, carcinogenicity) requires a paradigm shift concerning the information provided and use in risk assessment. The reason for this is that traditional approaches (animal studies) and NAMs have their strengths and weaknesses in different domains, and they can therefore not be translated 1:1. Animal studies generate the so-called apical endpoints, i.e., phenotypic changes that can be relatively directly related to human pathologies (Blaauboer 2012). Examples of this are tumour formation, morphological malformations, weight loss, anemia, or obvious histopathological alterations. These findings can be correlated with external doses that are administered by oral, dermal, or respiratory exposure. Very little information is available from standard animal studies on internal doses, organ concentrations of toxicants, and on the mechanisms leading to the observed changes.

By contrast, NAMs are best at providing relevant target site concentrations and indicating the pathways leading to pathologies. In most cases, they do not give direct information on pathological outcomes, and the NAMs data are difficult to relate to external doses and to various exposure situations. Two fundamental strategies have been developed which work together to bridge the gap between the types of data provided by NAMs and the type of data used for regulation: the adverse outcome pathway (AOP) concept and in vitro to in vivo extrapolation (IVIVE) (Terron et al 2018; Punt et al 2020; Bos et al. 2020; Kisitu et al. 2020).

AOPs describe how a molecular initiation event (MIE, for example, binding of a toxicant to its target) relates to a final adverse outcome (AO, apical endpoint). It is assumed that there is a defined chain of necessary key events (KE, mechanistic steps), and that measurement of the MIE or KE would allow an extrapolation to the AO. This concept allows the use of NAMs to identify and quantify MIEs and KEs to predict final pathologies relevant for risk management and regulation (Leist et al. 2014, 2017). More than 200 AOPs have been registered by now in the OECD data base, and some of them have been accepted for regulatory purposes (e.g., on dermal sensitization or endocrine disruption) (Kolle et al. 2020; Browne et al. 2020).

IVIVE is a procedure using toxicokinetic models to translate threshold concentrations found in NAMs to threshold or reference doses and corresponding exposures in animals and man. Two core elements are required for IVIVE. First, a physiologically based kinetic model (PBK, sometimes also called PBTK or PBPK in toxicology or pharmacology) needs to be developed. This is a mathematical model that predicts the concentrations of a toxicant (ideally also its metabolites) for any time and for any position in the body. It takes into account physiological parameters such as blood flow, organ sizes, or membrane transporters, but it also uses compound-specific parameters, such as xenobiotic metabolism (e.g., in the liver), plasma protein binding or renal elimination. The second big step is PBK model parameterization—generally run in parallel with the setup (e.g., providing data on hepatocyte metabolism or plasma protein binding). Many NAMs have been developed for this purpose. Both the model setup and the parameterization also play an important role in other fields, such as clinical pharmacology (Paini et al. 2019, 2021), human biomonitoring, and risk assessment (Louro et al. 2019). This field is thus already highly sophisticated and still developing and for this reason is not yet fully accepted into regulatory science but OECD have produced a guidance document on the characterisation, validation, and reporting of Physiologically Based Kinetic (PBK) models for regulatory purposes (OECD 2021b). Like many NAMs, there will be further developments which leads to the question of when application should take place. We would argue that bringing these methods into use will drive investment, and increase experience as well as confidence and their further development. It would therefore be expedient to use these methods now.

As already argued above, many NAMs are ready for their application in next-generation risk assessment (NGRA) (Moné et al. 2020; Dent et al. 2018, 2021) and for a transformation towards more flexible and more efficient risk evaluation approaches. However, there are also some issues to be clarified before a more wide-spread and radical implementation. The three most important ones are validation, quantification, and coverage. Validation refers to the process of assessing the prediction and accuracy of a method or strategy. It has been identified as a key factor to provide confidence to regulators and other stakeholders that the new approach is at least as protective as the traditional approach. Initial NAMs have been validated by conservative approaches using a 1:1 comparison to traditional methods. As this is not possible for modern NAMs, there is an intensive discussion presently ongoing on validation strategies (Leist et al. 2012b; Patterson et al. 2021; Bal-Price et al. 2018; Aschner et al. 2017; Marx et al. 2020). This also includes the second open issue: quantification (Spinu et al. 2020). Most AOPs are currently not yielding quantitative predictions, and IVIVE often comes along with large uncertainties. Experience and case studies run by industry or mixed research consortia need to clarify the relative uncertainties of new and traditional approaches such as the National Academies of Science review of the variability and relevance of existing laboratory mammalian toxicity tests for human health risk assessment to inform the validation and establishing scientific confidence in using NAMs (NAS 2021). And the same applies to the last, but not the least, important point of the three open issues: coverage. This term refers to uncertainties on whether NAM-based approaches cover all potential toxicants, i.e., whether they are sensitive enough to provide human safety. The potential gaps most frequently discussed are safety issues due to metabolites (that fail to be generated), pathologies due to MIE not included in a test battery, and chronic pathologies arising only after long-term exposure and possibly not detected in NAM-based short-term tests.

At present, it is not clear whether these concerns really limit the practical application of NAMs, and whether the uncertainties of NAMs are larger than those of animal-based approaches (e.g., problems of species extrapolation). Comparative studies used within a regulatory context will help to clarify this, and the parallel use of traditional and new approach methods for a transition period may be a practical approach to provide all required information (Lanzoni et al. 2019).

## Moving away from mandated study lists to mandated hazard categories and safe limit doses

Safety decisions are in general taken from two standpoints:

- Is this chemical safe to use in this situation?
- In which situations is this chemical safe to use?

From both standpoints, it is necessary to know something about both the hazard and the exposure. Sometimes, this is specific and sometimes it is generic. Classification lends itself to generic situations, such as the example quoted earlier of STOT category 1 chemicals not being allowed to be used for non-professional users. In this case, enough is known about the potential exposure to non-professional users that chemicals with the potency of STOT category 1 chemicals would be likely to exceed their safe dose. However, when the chemicals are planned to be used by professional users, a risk assessment is carried out in which the specific exposure is estimated and compared with the safe dose for the chemical. In these two cases, different levels of precision are required for both the hazard and the exposure to make the safety-based decision.

Currently, within REACH, there are tiered requirements for studies which are based on annual tonnage as a surrogate for exposure. The studies are then used to derive hazard classifications and safe doses. Could the requirements be stated in terms of which hazard categories or safe dose levels are required, rather than the studies which are required to generate them based on annual tonnage? In this way, information from lower tier studies could prove to be sufficient for some situations/uses. In other cases, higher tier studies would be needed. The examples in “Examples of the tiered approach hazard and exposure framework” illustrate these situations.

In addition, mandating particular studies tends to ossify the process and makes it very difficult to incorporate scientific and technological advances. Mandating hazard categories and safe dose levels would allow methodology to develop as long as it met performance standards, which is an approach taken in some situations by the European Medicines Agency (EMA) (Cave et al. 2020; EMA 2021).

Existing general chemicals have a range of uses which depends on the chemistry. The uses of the chemicals will dictate the desirable hazard categorisation and safe dose profile necessary to assure safety in the range of uses. The information which is available for the chemical can then be assessed using the tiered approach and a judgement could be made on whether any additional information is required. The tiered approach can then be used to decide the most effective way to generate the information and to assess safety. It would be the responsibility of the registrant to demonstrate that they have defined the appropriate hazard categorisation and safe dose profiles and that the chemical meets them. Precedent is set by the Cosmetics Directive where this is already the case.

Existing chemicals can also be considered for new uses. The first step in the safety assessment should be to determine the required hazard categorisation and safe dose profile required for the use. The chemical’s existing hazard and safe dose profile can then be assessed against what is required for the new use and a decision made as to whether it is acceptable, requires more information, or is unacceptable.

New chemicals will start off with a relatively narrow range of proposed uses. The required hazard categorisation and safe dose profile can be determined from considering those uses. The tiered approach can then be used until it is determined whether the chemical is safe or not for the proposed uses. In general, chemicals which are restricted to low exposure situations will not progress as far through the tiered approach as those with higher exposure potential uses; but this will be guided by the use and associated actual exposure not by the tonnage. As new uses are considered, the same process can be used.

## Examples of the tiered approach hazard and exposure framework

The utility and practicality of the tiered approach has been assessed by a series of case studies. In each case, a chemical has been selected which has both extensive NAMs data and traditional data. The process of each evaluation started with deciding on some uses for the chemicals, which were not necessarily the ones it is actually used for, designed to cover both industrial and consumer situations and to give a range of potential exposure doses.

The exposures were then estimated using the Targeted Risk Assessment (TRA) tool (ECETOC 2021b), which takes a range of uses and produces conservative estimates of exposure for a range of scenarios based on accepted models and using physicochemical data for the chemical. Higher tier exposure modelling using Advanced REACH Tool (Fransman et al. 2011) or ConsExpoWeb model (RIVM 2018) was performed to refine exposures, where necessary. These exposures were then categorised as explained in the section on Exposure Categorisation.

Once this had been done, the hazard assessment process commenced guided by these exposure categories. Each chemical was viewed as an unknown and in silico, in vitro, and in vivo information was used as if it had been commissioned by a registrant. The process was taken through to an appropriate place to stop once enough information had been obtained to make a decision on safety for the required uses. The outcome was compared with the outcome which would have been reached using conventional data.

The outcome from each tier was expressed in terms of the key adverse effects, the classification, and the DNEL. The current CLP hazard categories for carcinogenicity and for reproductive toxicity are based on the strength of evidence for the presence of the hazard rather than on degree of hazard and therefore pose a problem. This makes it difficult to operate a scheme which incorporates degree of hazard (severity and potency) as part of the process. For this exemplification, we have used the scheme suggested by Doe et al (2021). This uses the consideration of potency for carcinogenicity and for reproductive/developmental toxicity which are contained in the CLP guidance in determining specific concentration limits for preparations containing substances classified for these hazards. These were developed because of the concerns expressed by an expert EU working group (EC 1999) for carcinogenicity, that “the general classification system for carcinogens does not take into account the wide range of carcinogenic potency that can be observed both in human epidemiological studies and in animal experiments. As well as the need for a system to reflect this wide range of carcinogen potencies, there are examples of carcinogens where the question of potency as such is of particular concern”. Similar concerns were addressed for reproductive toxicity (ECHA 2017). Guidance is given on the assignment of chemicals classified for carcinogenicity or reproductive/developmental toxicity into high, medium, or low potency categories. We have used this guidance to categorise chemicals in these examples with high potency resulting in category 1, medium potency into category 2, and low potency into category 3.

Summaries of the examples are included in the paper. The full evaluations are available as Supplementary data.

### Example chemical 1 (EC1)

*Exposure assessment tier 1:* The uses of EC1 were selected to include both industrial and consumer use, and then, relevant exposure scenarios from the ECETOC TRA were selected. The relevant physicochemical data were entered into the TRA and the exposures estimated using worst-case assumptions:

- Use as a chemical intermediate: Category B.
- Use in metal working fluids: Category B.
- Use in glues for hobby use: Category B.

*Hazard assessment:* Tier 0: The exposures were above the TTC limits.

Tier 1 based on in silico assessment: EC1 would not be genotoxic on the basis of absence of alerts from the Derek Nexus software (Lhasa Ltd 2021).

Tier 2 Hazard assessment based on in vitro assays for biological activity, genotoxicity, absorption, and metabolism:

Non-genotoxic

MoAs identified and adverse outcomes predicted:

- Androgen receptor blocker—male reproductive toxicity leading to infertility, hypospadias, delayed development, and Leydig cell tumours.
  - LOEL 2 mg/kg NOEL 0.6 mg/kg from in vitro assays and IVIVE.
- Mitochondrial toxicity—acute lethality, liver toxicity.
  - LOEL 6 mg/kg NOEL 2 mg/kg from in vitro assays and IVIVE.
- CYP induction: liver enlargement, potential carcinogenicity.
  - LOEL 6 mg/kg NOEL 2 mg/kg from in vitro assays and IVIVE.
- PPAR: liver enlargement, peroxisomes, potential carcinogenicity.
  - LOEL 6 mg/kg NOEL 2 mg/kg from in vitro assays and IVIVE.

The hazard categories and DNEL are shown in Table 3.

**Table 3:** Comparison of outputs from tier 2, tier 3, and conventional hazard assessments for ECI

	Tier 2—in vitro	Tier 3—targeted in vivo	Conventional studies
STOT-RE category	1	1	1
Reproductive Tox category	1	2	2
Carcinogenicity category	1	2	2
DNEL	0.006 mg/kg	0.04 mg/kg	0.024 mg/kg
Number of animals used	0	100	2200

**Fig. 3:**
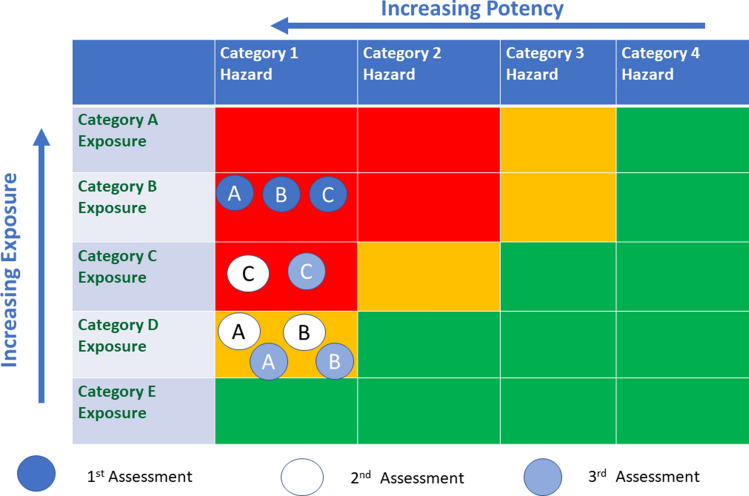
Use of the exposure and hazard matrix for EC1. Circles show position in the matrix after each assessment. Use **A** Chemical Intermediate; Use **B** Metal working fluid; Use **C** Hobby Glue. 1st Assessment Tier 1 Exposure/Tier 2 Hazard; 2nd Assessment Tier 2 Exposure /Tier 3 Hazard; 3rd Assessment Tier 2 Exposure/Conventional Hazard

*First assessment of hazard and exposure:* The consideration of exposure and of hazard showed category 1 hazard and category B exposure for all of the uses placing them in a red segment indicating unacceptable for use (Fig. 3).

*Refinement of exposure assessment:* Exposure assessment was refined to assume reasonable exposure controls in occupational settings (e.g., ventilation to reduce vapour exposure and to eliminate aerosols). Use as a chemical intermediate stays in Category B and use in metal working fluid and in hobby glue move from Category B to Category C. All uses remain in the red segment where exposure is above the DNEL (Fig. 3).

Tier 2 Exposure Assessment used the ART exposure model (Advanced REACH Tool, Fransman et al. 2011). The use of this model moves use as an intermediate and in metal working fluid to Category D but use in hobby glue remains in Category C (Fig. 3).

*Second assessment of hazard and exposure:* Hobby glue remains in a red segment where the options are to conclude as not suitable or progress to higher tiers. Use as a chemical intermediate and in metal working fluid move to an amber segment on the matrix which requires further consideration, including comparison of exposure and DNEL assessments. The DNEL from Tier 2 is 0.006 mg/kg. The exposures assessments from Tier 2 are 0.004 mg/kg for use as a chemical intermediate and 0.002 mg/kg for use in metal working fluid. There is not a sufficient level of confidence in a Tier 2 Hazard assessment to assure safe use so the option is to move to higher tiers.

*Tier 3 hazard assessment:* Following the Tier 2 assessment, anti-androgenicity and liver toxicity were identified as key modes of action. It was decided to investigate these using targeted in vivo studies in Tier 3.

- Anti-androgenicity was assessed with a Hershberger assay. EC1 showed anti-androgenic effects with an NOEL of 25 mg/kg.
- A rat 90 day study was performed with EC1. The effects seen were liver hypertrophy, adrenal hypertrophy, and Leydig cell hypertrophy with an NOAEL of 4 mg/kg/day.

MoAs identified and adverse outcomes predicted:

- Androgen receptor blocker—male reproductive toxicity leading to infertility, hypospadias, and delayed development. NOEL 25 mg/kg. The NOEL effect level in multigeneration studies is predicted to be the same as for the in vivo assay based on reference to other chemicals acting on same AOP assessed in multigeneration studies as described by OECD (2009). EC1 would be of medium potency for reproductive toxicity and thus be in hazard category 2.
- CYP induction leading to liver hypertrophy and possibly tumours. Anti-androgenicity leading to Leydig cell hyperplasia and possibly to tumours.
- CYP induction leading to liver hypertrophy and tumours, and adrenal hypertrophy. EC1 would be of medium potency for carcinogenicity and thus be in hazard category 2.

The hazard categories and DNEL are shown in Table 2.

*Third assessment of hazard and exposure:* Hobby glue remains in a red segment (Fig. 3) and it is prudent to conclude as unsuitable for use. Use as an intermediate and in metal working fluid remain in an amber segment on the matrix (Fig. 3) which requires further consideration, including comparison of exposure and DNEL assessments. The DNEL derived from Tier 3 assessment is 0.04 mg/kg. The exposures’ assessments from Tier 2 are 0.004 mg/kg for use as a chemical intermediate and 0.002 mg/kg for use in metal working fluid. There is enough margin of exposure and confidence in the hazard and exposure estimates to conclude that these uses would be safe provided that the risk management measures assumed in the exposure assessment are in place.

*Comparison with conventional assessment:* EC1 is vinclozolin which as a plant protection product has been the subject of a full conventional assessment. These are the relevant outcomes:

- In the rat multigeneration reproductive toxicity study, there were hypospadias and decreased ventral prostate weights at 50 mg/kg and above. There was reduced sperm count at 100 mg/kg. NOAEL 2.5 mg/kg.
- There were increased incidences of Leydig cell and adrenal tumours in the rat long-term bioassay with an NOEL of 2.7 mg/kg/day,
- There were increased incidences of liver tumours in mouse long-term bioassay with an NOEL of 24 mg/kg/day.

The hazard categories and DNEL are shown in Table 2.

*Comparison of hazard outputs*—The outputs from the Tiers can be compared in Table 2. The main adverse outcomes were identified in Tier 2 based on in vitro data. The classification categories were changed to become less severe for carcinogenicity and reproductive toxicity in tier 3. The DNEL increased by a factor of 10 moving from in vitro to in vivo but barely moved from targeted in vivo studies to full conventional studies. This is consistent with the findings of Friedman et al (2019) that the in vitro derived PoD has a measure of conservatism (up to 2 orders of magnitude) within it. The decision to proceed to tier 3 would depend on the proposed uses. If the margin of exposure were large enough, then there would be no need. The DNEL derived from a short-term focussed in vivo study was the same as for the conventional study.

### Example chemical 2 (EC2)

*Tier 1 usage and exposure assessment—*The uses of EC2 were selected to include industrial and consumer use and the relevant exposure scenarios from the ECETOC TRA were selected. The relevant physicochemical data were entered into the TRA and the exposures estimated using worst-case assumptions: possibility of aerosol, no modifying factors such as gloves, 100% absorption via inhalation, and by dermal routes. Each exposure was assigned a category:

- Uses requiring drum and small package filling (liquid or solid in liquid) Category A.
- Use in consumer cleaning PC35: dish washing products, Laundry products, all purpose cleaners, and Trigger Sprays Category A.

#### Hazard assessment

Note: The hazard data for this evaluation are extracted from Baltazar et al (2020).

*Tier 0*—All of the exposures are in category A; therefore, the TTC does not apply.

*Tier 1*—Based on in silico assessment. EC2 is an aromatic organic chemical compound classified as a member of the benzopyrone family. The in silico tools predicted that EC2 can bind to proteins and DNA via Michael addition and acyl transfer mechanism. Similarly, the OECD QSAR Toolbox predicted binding to DNA and proteins via SN2 mechanisms after oxidation to epoxide. No positive results were obtained from the MIE ATLAS tool (2021).

Hydroxylation was identified as the main route of biotransformation followed by glucuronidation and sulfation with a total of 22 possible metabolites. Most primary, secondary, and tertiary metabolites were predicted to bind to proteins and DNA.

In summary, these in silico alerts indicated a need to investigate the genotoxicity potential of EC2 and its metabolites.

Tier 1 assessment indicated the potential for some adverse effects, but could not provide an estimate of potency. Given that exposures are in Category A or B, it was necessary to move to Tier 2 Hazard Assessment.

*Tier 2 hazard assessment*—Based on in vitro methodology. In vitro assessment is a three-part process. Part 1 is determining what biological activity the chemical may have using a range of in vitro alerting assays. Part 2 is to use more specific assays to follow up on the activity indicated by part 1. Part 3 is to bring in assays and models based on kinetics and metabolism to provide an estimate of in vivo points of departure.

Summary from the tier 2 Hazard assessment is:

- Non-genotoxic.
- MoAs identified and adverse outcomes predicted:
- Monoamine oxidase inhibition PoD—12–40 µM.
- CYP 450 induction pathways—possible liver enlargement—PoD 6–60 µM.
- Carbonic anhydrase inhibition—PoD 21–62 µM.
- Cellular Stress panel of markers in HepG2 cells—500–800 µM.
- No other pathways identified.
- PoD range 6–60 µM.
- IVIVE—in vivo PoD = 36–360 mg/kg.

#### Output from tier 2 hazard

- Predict mild non-specific toxicity with liver hypertrophy/hyperplasia which may lead to hepatocarcinogencity at high doses.
- NOEL = 36–360 mg/kg.
- The hazard categories and DNEL are shown in Table 3.

*Overall assessment after tier 1 exposure and tier 2 hazard assessment—*The consideration of exposure and of hazard shows category 2/3 hazard and category A exposure for both uses placing them in a red to amber segment. The options are to decide EC2 is not suitable for those uses or to refine exposure or hazard assessment. As exposure is Category A, it was decided to refine exposure.

*Refine Tier 1 exposure assessment—*Adjustments were made in the TRA calculations for reasonable exposure control measures to be made for drum filling and using specific consumer exposure determinants (SCEDS AISE 2015). Drum filling in manufacture moves to Category C, but household uses remain in Category A.

**Fig. 4:**
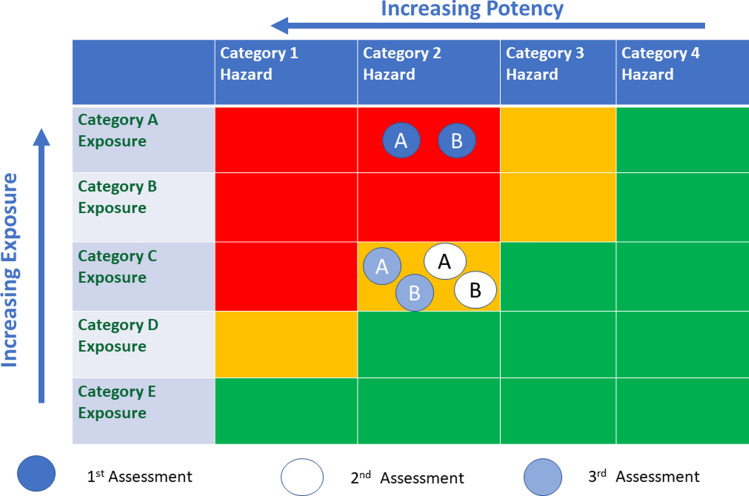
Use of the exposure and hazard matrix for EC2. Circles show position in the matrix after each assessment. Use **A** Household cleaning; Use **B** Drum filling. 1st Assessment Tier 1 Exposure/Tier 2 Hazard; 2nd Assessment Tier 2 Exposure/Tier 2 Hazard; 3rd Assessment Tier 2 Exposure/Conventional Hazard

*Overall assessment after tier 1 refined exposure and tier 2 hazard*—The uses are shown on the Exposure/Hazard Matrix (Fig. 4). Drum filling moved to an amber/green segment where it is prudent to compare exposure and DNEL. The exposure was calculated to be 0.5–1.45 mg/kg per day and the DNEL was calculated to be 0.36–3.6 mg/kg/day. Use in drum filling is borderline acceptable. Household cleaning uses remain Category A placing the use in a red/amber segment, so it was decided to move to Tier 2 exposure assessment.

*Tier 2 exposure assessment*—Exposure for drum filling was refined using the Advanced REACH Tool exposure model. Exposure for household use was refined using higher tier ConsExpoWeb model predicted exposure (RIVM 2021). All uses were in category C.

*Overall assessment for tier 2 exposure and tier 2 hazard* (Fig. 4)—The overall assessment for drum filling as borderline acceptable did not change as a result of tier 2 exposure assessment. Household use was placed in an amber/green segment where it is prudent to compare exposure and DNEL. Exposures ranged from 0.09 to 3.2 mg/kg/day and the DNEL was calculated to be between 0.36 and 3.6 mg/kg/day, also indicating borderline acceptable use.

*Comparison with conventional assessment—*EC2 is coumarin which has been the subject of conventional toxicology studies.

*Output from conventional animal studies—*2 year rat study: mild non-specific toxicity with hepatoenlargement. Hepatocarcinogencity at high doses.

NOEL = 16–42 mg/kg DNEL derived by application of uncertainty factor of 100.

The hazard categories and DNEL are shown in Table 4.

**Table 4:** Comparison of outputs for Tier 2 and conventional studies for EC2

	Tier 2—in vitro	Conventional studies
STOT-RE category	None	None
Reproductive Tox category	N/A	N/A
Carcinogenicity category	2	2
DNEL	0.36–3.6 mg/kg	0.16–0.42 mg/kg
Number of animals used	0	1000

*Overall assessment for tier 2 exposure and conventional hazard—*Both uses were placed in an amber segment where it is prudent to compare exposure and DNEL. Drum filling exposure was 0.5–2.1 mg/kg/day with DNEL calculated to be 0.16–0.42 mg/kg/day, borderline acceptable.

Household use exposures ranged from 0.09 to 3.2 mg/kg/day and the DNEL was calculated to be 0.16–0.42 mg/kg/day, also indicating borderline acceptable use.

*Comparison of hazard outputs*—The outputs from Tier 2 based on in vitro data and from the conventional assessments are similar in terms of the nature of the effects, the classification categories, and DNELs. This indicates that it is not possible to assume that the in vitro-,based DNEL will always be more conservative than the in vivo-based DNEL.

### Example chemical 3 (EC3)

A third chemical was evaluated for hazard, but no exposure assessment was made.

Data were extracted from an OECD IATA case study (OECD 2021c).

Tier 0—As a hazard evaluation was done, the use of the TTC was inappropriate.

#### Tier 1: in silico assessment

EC3 is a preservative which can inhibit malate dehydrogenase in bacteria, which is part of the citric acid cycle and therefore conserved across most, if not all, species including humans. Sequence homology was used to determine the level of sequence identity and similarity between the bacterial and human enzymes using CDOCKER module in Discovery Studio 2018 (ALTEM 2021).

In silico tools were also used to predict the likely metabolism of EC3.

The major metabolite was predicted using Meteor Nexus version 3.1.0 (Lhasa Ltd.) and also assessed using the in silico tools with the same results as for EC3.

#### Tier 2: in vitro assessments

A range of in vitro assays was performed: SafetyScreen44™, Cell Stress Panel in HepG2 cells and Transcriptomics in MCF7, HepG2 and HepaRG cells.

Summary from the tier 2 hazard assessment is

- Non-genotoxic.
- MoAs identified and adverse outcomes predicted:
  o No clear mode of action has emerged and EC3 should be considered to show general toxicity which is likely to be seen in the liver and/or kidneys.
- PoD range EC3 171–557 µM. Phenoxyacetic acid (major metabolite) 217–359 µM.
- IVIVE—In vivo PoD = 1.9–3.1 mg/kg.

Output from tier 2 hazard

- Non-specific toxicity, possibly liver toxicity.
- NOEL = 1.9–3.1 mg/kg
- DNEL derived by application of uncertainty factor of 100.

The hazard categories and DNEL are shown in Table 5.

*Comparison with conventional assessment: 90-day study in rats*

EC3 is phenoxyethanol.

- No specific toxicity. Liver weight increased, kidney, and bladder effects. Red blood decreases.
- NOAEL: male 369 mg/kg female 652 mg/kg.

*Output from conventional hazard assessment:*

- Non-specific toxicity, possibly liver toxicity.
- NOEL = 360–650 mg/kg.
- DNEL derived by application of uncertainty factor of 100.

The hazard categories and DNEL are shown in Table 4.

In this case, the Tier 2 evaluation produced a similar qualitative assessment, but indicated a lower DNEL than the conventional studies reflecting the overall tendency for in vitro-based DNELs to be more conservative than in vivo-based DNELs. The decision as to whether Tier 3 studies would have been carried out would have depended on the proposed usage and the resulting exposure.

The information could have been used for decision-making purposes by deeming that EC3 would be suitable for use in situations which result in Category C, D, and E exposures by reference to the exposure range for longer term use, as shown in Table 2.

**Table 5:** Comparison of Tier 2 and conventional assessments for EC3

	Tier 2—in vitro	Conventional studies
STOT-RE category	None	None
Reproductive Tox category	N/A	N/A
Carcinogenicity category	None	None
DNEL	0.02–0.03 mg/kg	3.6–6.5 mg/kg
Number of animals used	0	160

## Comments on examples

The first two examples illustrated the use of the framework to carry out a tiered evaluation of both exposure and hazard. The assessments of both hazard and exposure were refined until a decision could be taken that the proposed uses were either acceptable or unacceptable by reference to the matrix. The third example illustrated a hazard only assessment. At the end of the Tier 2 assessment, classification categories and a DNEL were available. Use of the exposure/hazard matrix indicated the exposure categories for which the chemical would have been suitable. This would allow product selection to go ahead.

The examples were chosen, because there were in vitro and in vivo data available which allowed the comparisons to be made. The cases showed a range of differences between the in vitro-based DNELs and the in vivo-based DNELs from parity to two orders of magnitude lower. This small sample suggests that it cannot be assumed that an individual in vitro-based DNEL is more conservative than an in vivo-based DNEL, although the overall trend would indicate that in vitro DNELs are more conservative (Friedman et al. 2019). The examples included a chemical with a clear potent mode of action, vinclozolin, and two chemicals with less specific toxicity, coumarin, and phenoxyethanol. The approach was shown to be applicable in both situations.

## The role of in vivo tier 3 studies

The tiered approach includes the use of in vivo studies, but this does not signal a reversion to the conventional menu-driven approach. If there is not the necessary level of confidence to make a decision based on the results of a tier 2 in vitro-based assessment, which could either be to approve or to disapprove a use, and then targeted in vivo studies can be designed. Their design will depend on the outcome of the tier 2 assessment. At this stage, it will be known whether the chemical has structural similarities with chemicals of known toxicity. Its activities in a wide range of assays for biological signals which lead to adverse effects will be known, its metabolism and kinetics profile will have been investigated, and assessments made of its classification and DNEL profile. It will also depend on the confidence in the evidence of absence of a particular toxicological potential.

The in vivo studies should be designed to test a hypothesis arising from the tier 2 assessment. The impetus to move to tier 3 will be driven in the main by two concerns:

- Is the potency seen in vitro going to be seen in vivo?
- Has an important adverse effect been missed?

The first question will require specific studies to be designed which investigate the in vivo potency. The studies can be focussed on an MIE or key biological event in the pathway which has a known relationship to the adverse effect. These studies can be of short duration, which must be long enough for the key event to occur and be observed. It is also helpful for extrapolation purposes to include toxicokinetics in the design of these studies.

The second question, has something been missed, is more open and requires a different approach. As experience grows with the use of in vitro-based methods, confidence in their use will increase. The results of in vitro methods should not be used on their own to make decisions. They should always consider the chemical structure and what is already known about it. A higher level of confidence would be appropriate for a chemical with a structural similarity to a well-studied group of chemicals that shows similar results in the in vitro studies. Chemicals which have no structural similarity to well-studied groups would have lower levels of confidence. Friedman et al. (2019) have shown that in vitro assessment based on screening for biological activity in a range of assays coupled with IVIVE extrapolation tends to provide more conservative points of departure than traditional in vivo studies. When the margin of exposure from the in vitro studies is low and the chemical structure is not well studied but no specific toxicity has been identified, then the decision could be to move to tier 3.

OECD guideline study 422 was designed for this situation. It provides a combined assessment of repeat dose toxicity and reproductive toxicity. It has been used as a screening study, but Beekhuijzen et al. (2014) evaluated the results of 134 studies and considered that it could be used as a definitive study to assess repeat dose toxicity and reproductive toxicity. The study could be revised in the light of developments in understanding the development of longer term outcomes such as carcinogenicity. The basic dosing regime of 4 weeks for toxicity and a single generation for reproduction should remain, but the observations in each phase could be revised to provide more information as knowledge increases.

Targeted in vivo studies can strengthen read-across comparisons where the biological signatures of chemicals are similar.

## Compliance of examples with REACH under Annex XI

There is provision within REACH for the use of NAMs within Annex XI where it states that factors can be used such as the use of existing data, weight of evidence, qualitative or quantitative structure activity relationships, in vitro methods, grouping of substances and read-across, and substance-tailored exposure-driven testing. Annex XI sets out the criteria for not requiring the mandated list of studies.

***Overall principle of tiered approach:*** Annex XI Sect. 1.2 states that “There may be sufficient weight of evidence from several independent sources of information leading to the assumption/conclusion that a substance has or has not a particular dangerous property, while the information from each single source alone is regarded insufficient to support this notion. There may be sufficient weight of evidence from the use of newly developed test methods, not yet included in the test methods referred to in Article 13(3) or from an international test method recognised by the Commission or the Agency as being equivalent, leading to the conclusion that a substance has or has not a particular dangerous property.”

This section provides the justification for the principle of integrating data in a tiered approach which we have used in developing the framework.

**In vitro studies:** Annex XI Sect. 1.4 provides criteria for the acceptance of in vitro studies:

“Results obtained from suitable in vitro methods may indicate the presence of a certain dangerous property or may be important in relation to a mechanistic understanding, which may be important for the assessment. In this context, ‘suitable’ means sufficiently well developed according to internationally agreed test development criteria [e.g. the European Centre for the Validation of Alternative Methods (EURL ECVAM at JRC)] criteria for the entry of a test into the prevalidation process). Depending on the potential risk, immediate confirmation requiring testing beyond the information foreseen in Annexes VII or VIII or proposed confirmation requiring testing beyond the information foreseen in Annexes IX or X for the respective tonnage level may be necessary.

If the results obtained from the use of such in vitro methods do not indicate a certain dangerous property, the relevant test shall nevertheless be carried out at the appropriate tonnage level to confirm the negative result, unless testing is not required in accordance with Annexes VII to X or the other rules in this Annex.

Such confirmation may be waived, if the following conditions are met:

(1) Results are derived from an in vitro method whose scientific validity has been established by a validation study, according to internationally agreed validation principles;

(2) Results are adequate for the purpose of classification and labelling and/or risk assessment; and.

(3) Adequate and reliable documentation of the applied method is provided.”

The in vitro methods used in the worked examples meet criterion 1. The use of a range of assays to detect biological activity coupled with in vitro assays and kinetic modelling to derive points of departure have been validated in an international collaboration including the European Chemical Agency, the EU Joint Research Centre, US Environmental Protection Agency, and Health Canada (Friedman et al. 2019).

The methods meet criterion 2, the major modes of action and adverse outcomes have been identified, and, together with determination of points of departure, they can be used for classification and categorisation and for the derivation of safe doses for risk assessment.

The evidence used and the logic for their integration into a weight of evidence approach is documented to meet criterion 3.

***In vivo studies:*** Annex XI Sect. 1.1.2. states the criteria for experiments not carried out according to GLP or the mandated test methods:

“Data shall be considered to be equivalent to data generated by the corresponding test methods referred to in Article 13(3) if the following conditions are met:

1. Adequacy for the purpose of classification and labelling and/or risk assessment;
2. Adequate and reliable coverage of the key parameters foreseen to be investigated in the corresponding test methods referred to in Article 13(3);
3. Exposure duration comparable to or longer than the corresponding test methods referred to in Article 13(3) if exposure duration is a relevant parameter; and
4. Adequate and reliable documentation of the study is provided.”

The in vivo methods used in the worked examples meet these criteria. For criterion 1, they provide confirmation of modes of action leading to adverse outcomes or adverse outcomes themselves which can be used for classification and the derivation of safe doses for risk assessment. For criterion 2, where studies are targeted at specific modes of action, the results in conjunction with the range of in vitro assays for biological activity cover the key parameters in the mandated studies. The broader ranging studies are designed to cover the key parameters. For criterion 3, the knowledge gained on the consequences of modes of action mean that it is not necessary for exposure to be for the same length of time as the mandated study for this criterion to be met. For instance, anti-androgenic activity is known to produce infertility over extended dosing and this can be assumed once the mode of action has been identified and characterised for potency. Induction of liver enzymes can be identified and characterised in studies of 2–4 weeks and the outcomes after 3 months or 2 years are known.

The evidence used and the logic for their integration into a weight of evidence approach are documented to meet criterion 4.

This section of Annex XI allows the use of studies not done to GLP or not performed to guideline protocols. It should be noted that these are different issues. Performing studies to GLP provides assurance on the technical integrity and reliability of the data. Studies not performed to guidelines should be carried out within GLP as far as possible. Justification for their use should also be provided.

Section 3 of Annex XI also allows for adaptation of standard information requirements listed in Annex VII to X based on exposure considerations.

This analysis of Annex XI was undertaken with the intention of finding reasonable justification for the use of NAMs in REACH. We have shown that it should be possible to interpret the legislation in ways which can support the increased use of NAMs in REACH. However, it is clear that it is also possible to interpret Annex XI in other ways which do not fully support the introduction of NAMs in the ways proposed in this paper. We acknowledge that the legislation makes it difficult to accept the use of NAMs in ways that do not replicate the mandated studies which form the basis of CLP. The authors of Annex XI may have assumed that new methods would be developed in the future that would address reduced use of laboratory animals but through a study for study replacement rather than the integration of different data sources, for which is there is now a growing consensus. To enhance the use of such new approaches, this needs to be addressed in the redrafting of REACH to facilitate the use of NAMs.

## Stepwise implementation

There is clearly not going to be a moment in time when new methodologies move from unproven to proven and the conventional laboratory animal-based approach will be stopped. Instead, there will be a process of evolution from the current situation where the majority of evaluation is done on the basis of the mandated studies approach with some exceptions to a situation where the majority of the evaluations are done using NAMs, including specifically designed in vivo studies, with some traditional in vivo studies. The pace of change will depend on building confidence through usage of the new methodology, initially in parallel with the traditional methods, and the status of the methodology in each hazard area.

There are established schemes for using NAMs in skin and eye irritancy/corrosion (Alepee et al. 2019; OECD 2017), dermal sensitization (Jaworska et al. 2013; Natsch et al. 2021), and mutagenicity (Petkov et al. 2015; Corvi and Madia 2017) which can be used now with reasonable confidence. There are in silico, in vitro, and focussed in vivo methods for acute toxicity which can also be used now with confidence (Erhirhie et al. 2018; Firman et al. 2022). NAMs for repeat dose toxicity are being actively developed and as discussed earlier should be judged on a case by case basis. The output from repeat dose toxicity and mutagenicity evaluations can be used for assessment of carcinogenicity. Furthermore, an increased understanding of the aetiology of cancer is giving rise to questioning the way hazard assessment for cancer is performed (Wolf et al. 2019). As more factors are identified which can modify cancer risk such as lifestyle and obesity, the binary concept of dividing chemicals into carcinogens and non-carcinogens based on the results of epidemiology and chronic rodent bioassays is becoming more difficult to sustain as cancer is recognised to be the result of a stochastic process with no definitive line between cancer and non-cancer (Doe et al. 2019; Harrison and Doe 2021). At the moment, categorisation is done on the basis of the strength of evidence for hazard identification, i.e., how convincing is the evidence that there is a causal relationship between the chemical and cancer, not on degree of hazard, i.e., the dose and duration of dosing required to cause cancer. Our examples show that it would be possible to develop a tiered approach which could categorise cancer potential on the basis of degree of hazard at different dose levels and enable the determination of reference doses for risk assessment (Cohen et al. 2019; Doe et al. 2021). This would allow the current practice of mandatory downstream risk management measures to continue for those chemicals deemed to have the highest degree of hazard.

Reproductive and developmental toxicity studies use over 60% of the animals required to complete a full evaluation of a chemical (van der Jagt et al. 2004). Reproductive toxicity is usually the result of a specific mode of action which affects the reproductive organs directly or indirectly by endocrine changes. In these cases, there may be in silico and in vitro methods which can indicate that reproductive toxicity may ensue, but they have yet to be put together into an IATA. Developmental toxicity is less well understood than cancer and reproductive toxicity, but there are in vitro assays which have been developed, although none have been accepted as suitable to replace the in vivo assays. As with carcinogenicity, the current classification scheme is based on strength of evidence, not on degree of hazard (severity, potency, and reversibility). This would need to be amended to allow this approach to be used (Doe et al. 2021). It would then allow the current practice of mandatory downstream risk management measures to continue for those chemicals which had the highest degree of hazard.

The level of confidence in an evaluation also depends on the level of the exposure; the greater the margin of exposure, the greater the confidence. The concept of categorising exposures and comparing the exposure and hazard categories allows this to be quantified and visualised.

We would suggest a gradual introduction of NAMs to build confidence. The initial steps could be taken in situations where there are better developed methods of hazard assessment, or where the current tonnage-based requirements do not require extensive experimentation, or where exposure will be low.

## Discussion

The framework described in this paper is part of an ECETOC Transformational Programme that, over a 3–5 year timespan, addresses longer term issues of scientific relevance that have the potential to transform chemicals management. A clear consensus emerged from meetings with member companies and stakeholders from academia and regulatory agencies that the way chemical safety assessment is done needs to change from the system which was developed in the 1980s. The current system uses many animals, is expensive and time consuming and, while it has served society well, it is based on assumptions which were made over 50 years ago. Science and understanding have moved on and so must the regulatory paradigm.

The current framework has been successful in allowing the use of chemicals for the benefit of society without causing harm (Herzler et al. 2021) and there is a reluctance to change to a new process until it has been properly validated. We need to look back through the current animal-based methods that have been refined over the years of their use. Some have been abandoned such as the LD_50_. It is therefore unrealistic to consider that NAMs will be introduced as a ‘finished product’ or total replacement. Rather, we need to enter an active period of introduction and then refinement based on use. This is to a degree being driven by societal demands and associated legislation in some areas of prominence such as cosmetics. Where this driver is not in place, some conservative opinions prevail for understandable legal reasons that have also developed over the last 50 years and not always beneficially. There is a need to step forward and this step forward should be driven by the regulatory agencies and by public understanding.

Consultation with stakeholders provided clear guidance on what is required of a new system: it should allow science-based safety decisions to be made which provide the same level of safety using fewer animals, taking less time, and using less financial and expert resource but operating as far as possible within the existing regulatory framework.

This suggested to us that we could move things forward by developing a framework in which new methods could be used together as they emerged. The framework would become the environment in which scientific validity, operational efficiency, and concordance with regulatory decision-making would be the criteria for the development and adoption of new methods.

In this paper, a framework has been described which meets these criteria. Its key elements are tiered approaches to hazard and exposure assessment which provide regulatory decision-making outputs at each tier in the form of classification/categorisation, limit values (DNELs, ADI, etc.), and exposure estimates. These can then be used within existing regulatory and risk management schemes.

The examples we have evaluated demonstrate the workability of the process. The outcomes of the in silico and in vitro assessments have been qualitatively similar to the conventional assessments which would have been made and in two of the three cases are quantitatively more conservative. The parallel assessment of exposure allows decisions to be made about whether more information has been required which would necessitate targeted in vivo studies. The exposure categorisation based on the criteria for hazard categorisation allows such decisions to be made in a rapid and transparent way. The process can be made situation-specific risk assessment and provide generic guidance on the suitability of chemicals for potential use. It can also be used to specify the desired profile based on hazard category when considering a range of chemicals for a particular product or use.

We have exemplified the framework within the existing REACH processes, showing how NAMs could be used to provide the necessary outputs. The farsighted people who drew up the original REACH legislation foresaw the development of new methodology and made provision for its use in REACH Annex XI. The framework we have developed incorporates the factors which were outlined in Annex XI: the use of existing data, weight of evidence, qualitative or quantitative structure-activity relationships, in vitro methods, grouping of substance and read-across approach, and substance-tailored exposure-driven testing. We have shown that the worked examples could be considered to meet the specified acceptance criteria in Annex XI, but this would require a re-interpretation of the current legislation by many stakeholders and it may be preferable to redraft the legislation.

Fentem et al. (2021) stated that we are at a tipping point in the development of chemical safety assessment methodology. They pointed out that Article 25 of the REACH legislation states that “testing on vertebrate animals for the purposes of this Regulation shall be undertaken only as a last resort” (EC 2006). However, the use of animals has increased from an annual average of 270,000 in the first 10 years of REACH (Taylor 2018) to 2,395,056 in 2019 (ECHA 2021). This indicates that the provisions within REACH for the use of non-animal assessment methods may not yet be used to their full potential. Fentem et al. (2021) called for the joining of forces across policy makers, scientists, regulators, and lawyers, to lead the paradigm shift which would truly allow animal testing to be a last resort.

The application of the framework we have described and evaluated would allow a measured and phased introduction of new methodology in chemical safety assessment initially with more developed methods of hazard assessment, or with low tonnage chemicals or with low exposure situations. This would meet the goal of science-based safety decisions which provide the same level of safety using fewer animals, taking less time, and using less financial and expert resource whilst still operating within the existing regulatory framework. It would also allow new methodology to be incorporated as it develops to further improve through continuous selective breeding rather than periodic revolution.

Issues to be resolved

- How to validate methodology with respect to safety assessment. Can one anchor NAMs to their regulatory impact on classification and risk assessment, rather than their individual technical outcomes?
- How to deal with NAMs that do not substitute traditional methods 1:1 as stand-alone methods? Regulatory prediction derived from integrated methodology needs further validation.
- How to deal with heterogeneous coverage of toxicological endpoints by currently available NAMs. Methodology is less advanced in some areas (e.g., developmental toxicity) than in others (acute topical toxicity)? This should not stop methods being adopted in more advanced areas, while research efforts need to be re-focussed on areas lagging behind.
- How to use the vast amount of legacy data from the last 70 years for setup and validation of methods and for support of specific assessments?
- How to design, perform, and validate novel types of in vivo studies, which provide more information, using fewer animals, and require less time?
- How to better use opportunities within the existing legislation for the adoption of new methodology? Can policies of major regulatory agencies be adapted to further encourage the use of data from NAMs?

## Supplementary Information

Below is the link to the electronic supplementary material.

Supplementary file1 (PDF 852 KB)
