# Supplementary material for: A framework for chemical safety assessment incorporating new approach methodologies within REACH
Source: Arch Toxicol. 2022 Feb 1;96(3):743–66. doi: 10.1007/s00204-021-03215-9 (PMC8850243; doi:10.1007/s00204-021-03215-9)
Supplement: Supplementary file 1 — Supplementary file1 (PDF 852 KB) [file 204_2021_3215_MOESM1_ESM.pdf]

**Example Chemical 1**

**Physicochemical data**

IUPAC name: 3-(3,5-dichlorophenyl)-5-ethenyl-5-methyl-1,3-oxazolidine-2,4-dione

Canonical SMILES: CC1(C(=O)N(C(=O)O1)C2=CC(=CC(=C2)Cl)Cl)C=C

Physicochemical data were obtained from PubCHem
https://pubchem.ncbi.nlm.nih.gov/compound/Vinclozolin#section=Computed-Properties

**Tier 1 Usage and Exposure Assessment**

The uses of EC1 were listed and the relevant exposure scenarios from the ECETOC TRA ( https://www.ecetoc.org/tools/targeted-risk-assessment-tra/ ) were selected.

Use as a chemical intermediate – General exposure open systems. Chemical production where opportunity for exposure arises, including aerosol. TRA PROC4

Use in metal working fluids Industrial - General exposure open systems. Chemical production where opportunity for exposure arises, including aerosol. PROC4

Use in glues for hobby use; skin surface contact. Consumer PC1

The relevant physicochemical data were entered into the TRA and the exposures estimated using worst case assumptions: possibility of aerosol, no modifying factors such as gloves, 100% absorption via inhalation and by dermal routes.

Each exposure was assigned a category.

| Use | Inhalation mg/m$^3$ 8 hr/day | Dermal mg/kg/day | Total dose per day (assuming 16m$^3$ per day) | Duration | Exposure Category |
|---|---|---|---|---|---|
| Chemical intermediate | 0.9 | 7 | 7.8mg/kg | Long term | B |
| Metal working fluid 1-5% | 0.9 | 7 | 7.8mg/kg | Long term | B |
| Hobby glue at 0.3% | 0.00017 | 1.8 | 1.8mg/kg | Intermittent | B |

**Tier 0 Hazard Assessment**
EC1 is Cramer class III. The boundary for Category E exposure for Cramer class III is 1.5µg/kg/day so all of the exposures from Tier 1 are above this limit and the assessment needs to progress to Tier 1.

**Tier 1 Hazard Assessment**
Tier 1 hazard assessment is based on in silico assessment. In this case the assessment was limited to a prediction that EC1 would not be genotoxic from the DEREK NEXUS platform (https://www.lhasalimited.org/products/derek-nexus.htm ) and an oral LD50 of 1073.43mg/kg from the EPA TEST program ( https://www.epa.gov/chemical-research/toxicity-estimation-software-tool-test )

These estimates do not address the uses of EC1.

**Tier 2 Hazard Assessment**

Tier 2 Hazard assessment is based on in vitro methodology. In vitro assessment is a three part process. Part 1 is determining what biological activity the chemical may have using a range of in vitro alerting assays. Part 2 is to use more specific assays to follow up on the activity indicated by part 1. Part 3 is to bring in assays and models based on kinetics and metabolism to provide an estimate of in vivo effect and no effect levels.

Part 1; Information on EC1 was gained from the range of assays in the EPA ToxCast program (https://www.epa.gov/chemical-research/exploring-toxcast-data-downloadable-data ). The assays indicated:

- Antagonism of androgen receptors
- Induction of a range of p450 enzymes
- PPAR receptor activation
- Disruption of the mitochondrial membrane potential

In addition a battery of in vitro genetic toxicity assays were all negative.

Part 2; The information on androgen antagonism was further investigated using a yeast androgen screening assay (YAS) to determine a point of departure (POD) which was defined as 3x background activity..

Part 3; In vitro assays were performed to determine clearance based on microsome degradation data and absorption based on CaCo data. The parameters were then used in the Berkeley-Madonna Model (https://berkeley-madonna.myshopify.com/ )to determine an in vivo POD of 2.28mg/kg bw for androgen antagonism.

The output from the Tier 2 Hazard assessment is:

MoAs identified and adverse outcomes predicted:

- androgen receptor blocker - Male reproductive toxicity leading to infertility, hypospadias, delayed development, Leydig cell tumours
    - LOEL 2mg/kg NOEL 0.6mg/kg from in vitro assays and IVIVE
- mitochondrial toxicity – acute lethality, liver toxicity
    - LOEL 6mg/kg NOEL 2mg/kg from in vitro assays and IVIVE
- CYP induction: liver enlargement, carcinogenicity
    - LOEL 6mg/kg NOEL 2mg/kg from in vitro assays and IVIVE
- PPAR: liver enlargement, peroxisomes, carcinogenicity
    - LOEL 6mg/kg NOEL 2mg/kg from in vitro assays and IVIVE

In terms of classification and DNEL, EC1 would be:

Classification

- STOT RE Category 1
- Reproductive Toxicity Category 1*
- Carcinogenicity Category 1*

Long term and intermediate DNEL: 0.006mg/kg/day

**Overall assessment after Tier 1 Exposure and Tier 2 Hazard Assessment**

The three uses of EC1 are shown on the Exposure/Hazard Matrix. The consideration of exposure and of hazard shows category 1 hazard and category B exposure for all of the uses placing them in a red segment. The options are to decide EC1 is unsuitable for those uses or to move to the next tiers.

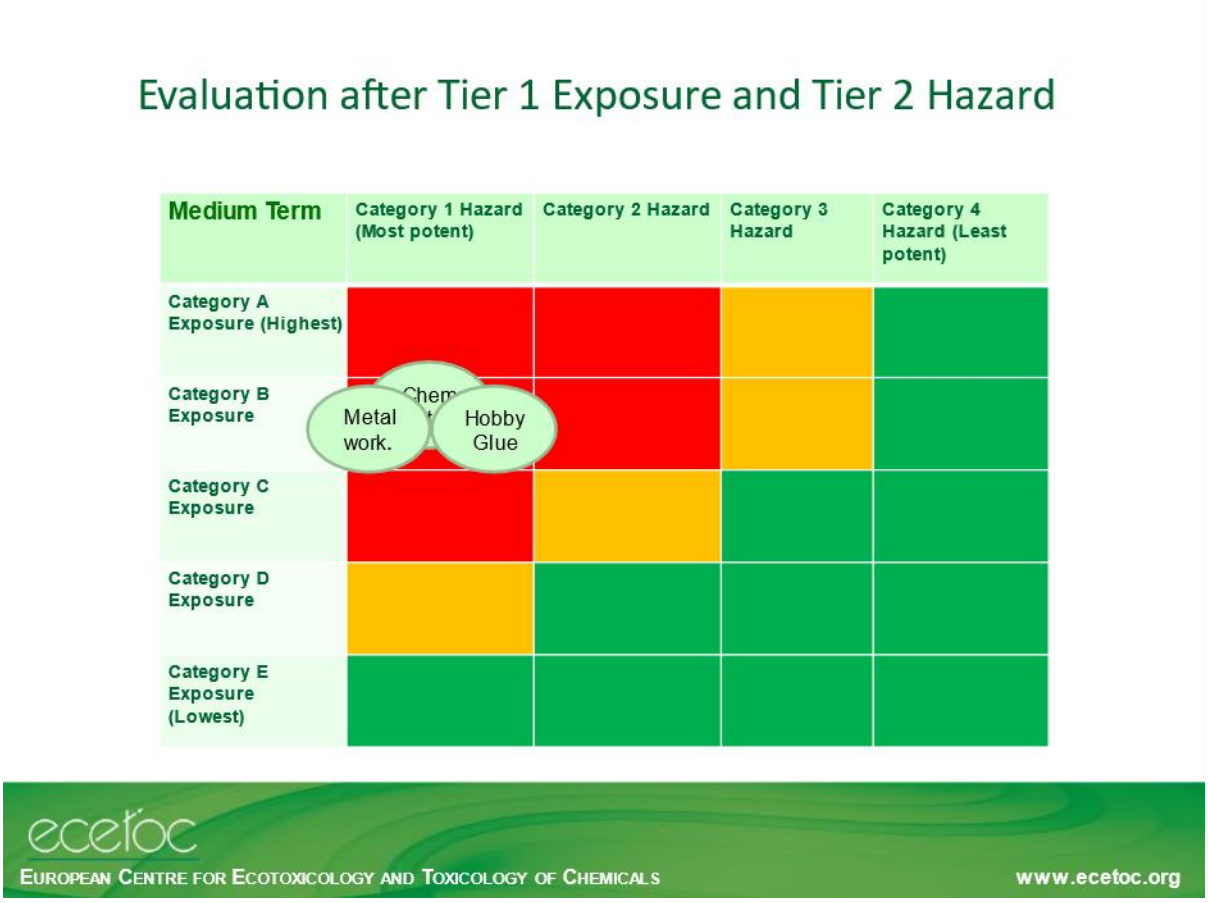


**Refine Tier 1 Exposure Assessment**

The first Tier 1 exposure assessment used worst case assumptions with no mitigation measures in place. The first refinement is to assume that there would be ventilation and protection to reduce vapour exposure and to eliminate aerosols.

- Use as a chemical intermediate – General exposure open systems. Chemical production where opportunity for exposure arises, no aerosol, ventilation, gloves. PROC4
- Use in metal working fluids Industrial - General exposure open systems. Chemical production where opportunity for exposure arises, no aerosol, ventilation, gloves. PROC4
- Use in glues for hobby use; skin surface contact. Revised estimate of deraml exposure. Consumer PC1

Those adjustments were made in the TRA calculations to arrive at the exposure estimations shown below.

| Use | Inhalation mg/$m^3$ 8 hr/day | Dermal mg/kg/day | Total dose per day (assuming 16$m^3$ per day) | Duration | Exposure Category |
|---|---|---|---|---|---|

| | | | | | |
|---|---|---|---|---|---|
| Chemical intermediate | 0.27 | 1.4 | 1.7mg/kg | Long term | B |
| Metal working fluid 1-5% | 0.13 | 0.27 | 0.4mg/kg | Long term | C |
| Hobby glue at 0.3% | 0.001 | 0.075 | 0.075mg/kg | Intermittent | C |

Use as a chemical intermediate stays in Category B and use as in metal working fluid and in hobby glue move from Category B to Category C.

**Overall Assessment after Tier 1 Refined Exposure and Tier 2 Hazard**

The three uses are shown on the Exposure/Hazard Matrix. All uses remain in the red segment where exposure is above the DNEL.

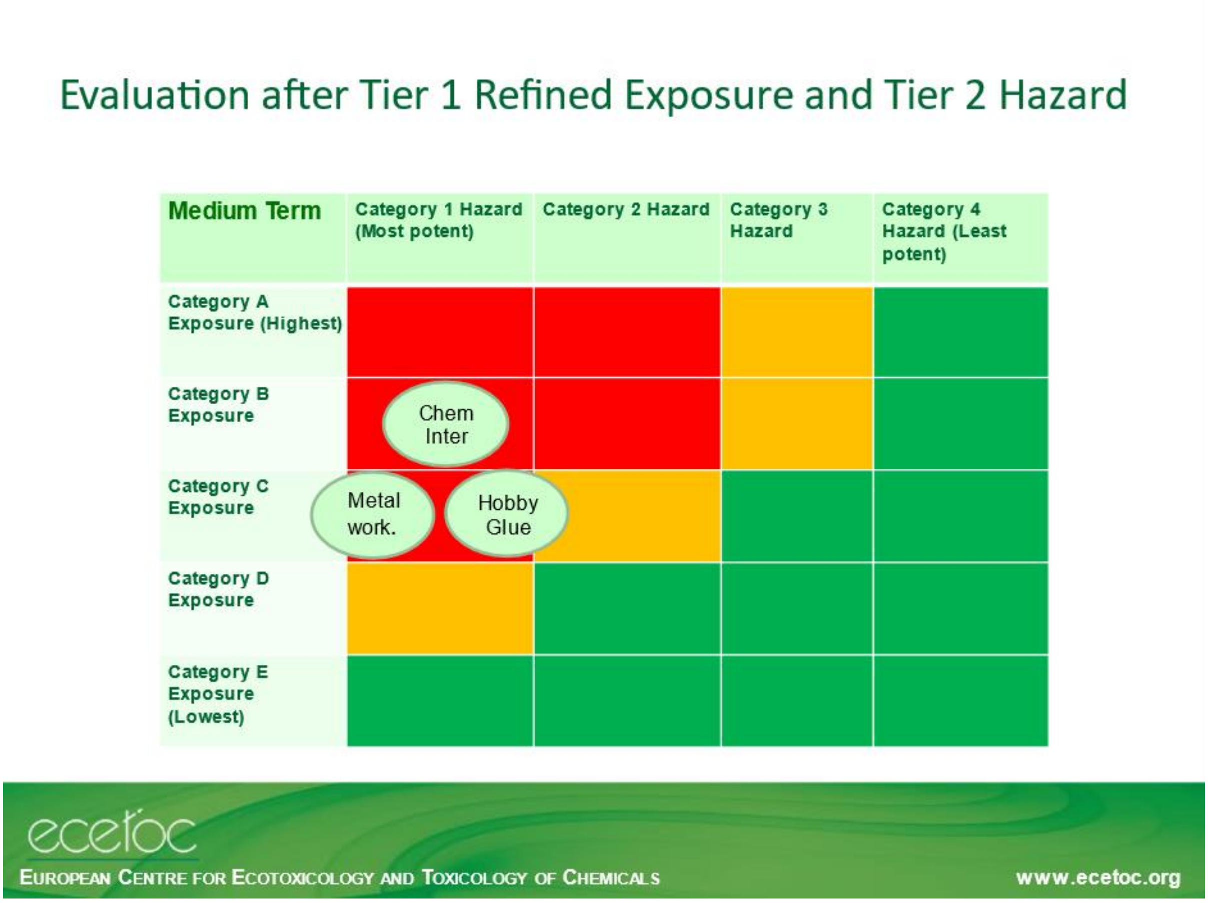


**Tier 2 Exposure Assessment**

Exposure was refined using the ART exposure model (Advanced REACH Tool - The Advanced Reach Tool - ART ). In this model, use as an intermediate and in metal working fluid were assumed to take place in well controlled work places with measures to minimise exposure.

| Use | Inhalation mg/m$^3$ 8 hr/day | Dermal mg/kg/day | Total dose per day (assuming 16m$^3$ per day) | Duration | Exposure Category |
|---|---|---|---|---|---|
| Chemical intermediate | 0.004 | 0 | 0.004mg/kg | Long term | D |
| Metal working fluid 1-5% | 0.002 | 0 | 0.002mg/kg | Long term | D |
| Hobby glue at 0.3% | 0.00017 | 0.13 | 0.13mg/kg | Intermittent | C |

The use of this model moves use as an intermediate and in metal working fluid to Category D but use in hobby glue remains in Category C.

**Overall Assessment for Tier 2 Exposure and Tier 2 Hazard**

Hobby glue remains in a red segment where the options are to conclude as not suitable or progress to higher tiers. Use as a chemical intermediate and in metal working fluid move to an amber segment on the matrix which requires further consideration, including comparison of exposure and DNEL assessments. The DNEL from Tier 2 is 0.006mg/kg. The exposures assessments from Tier 2 are 0.004mg/kg for use as a chemical intermediate and 0.002mg/kg for use in metal working fluid. There is not a sufficient level of confidence in a Tier 2 Hazard assessment to assure safe use so the option is to move to higher tiers.

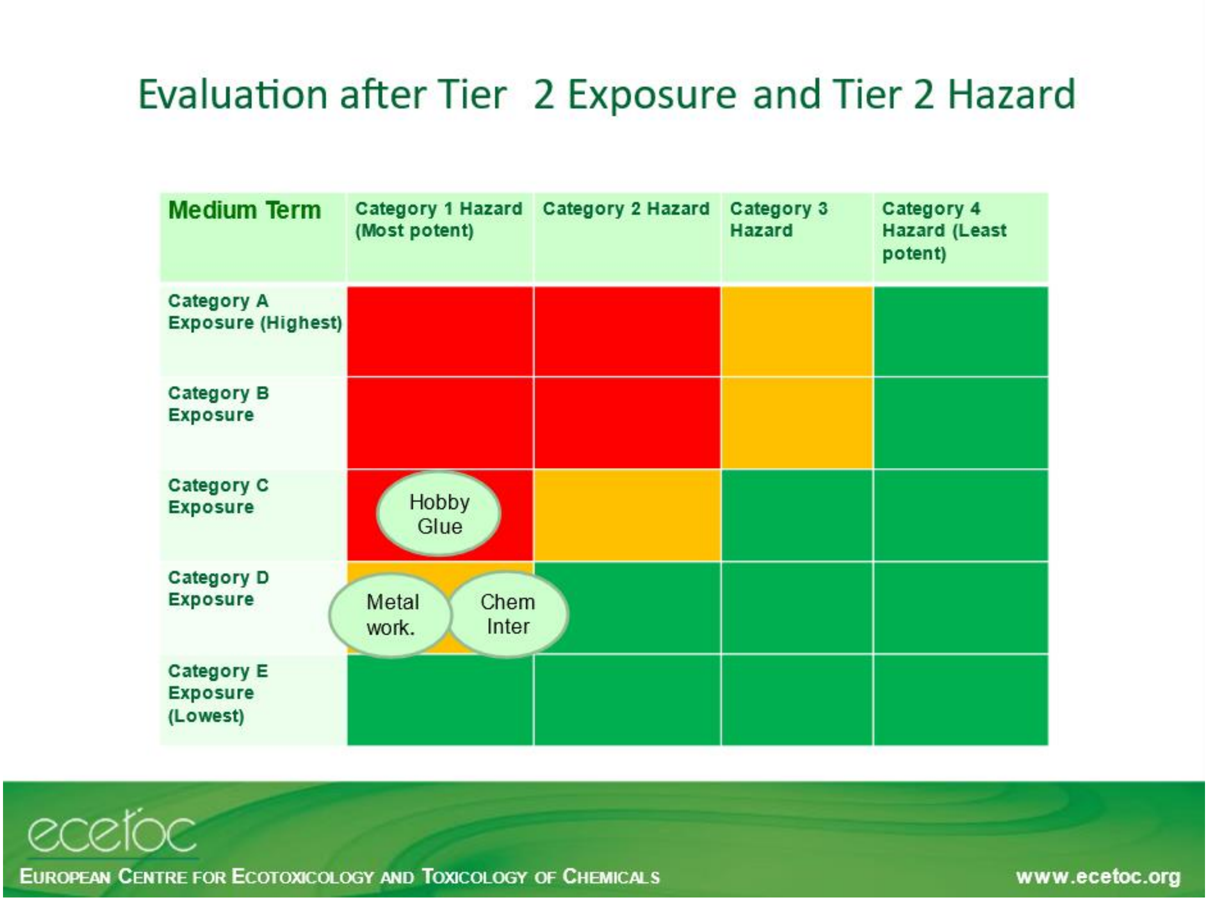

**Tier 3 Hazard Assessment**

Following the Tier 1 assessment, anti-androgenicity and liver toxicity were identified as key modes of action. It was decided to investigate these using targeted in vivo studies.

Anti-androgenicity was assessed with a Hershberger assay. The Hershberger assay is based on the principle that a number of organs and accessory sex tissues in the male reproductive tract require androgens to stimulate and to maintain growth. If the endogenous
source of this hormone is not available, either because of immaturity of the animals or because the animals have been surgically castrated, the animal requires an exogenous source to initiate and/or restore the growth of these tissues . Chemicals that act as agonists may be identified if they cause a statistically significant increase in the weights of the target androgen-dependent tissues, or chemicals may be identified as antagonists if they cause a statistically significant decrease in target tissues when coadministered with a potent androgen. EC1 showed anti-androgenic effects with a NOEL of 25mg/kg.

A rat 90 day study was performed with EC1. The effects seen were liver hypertrophy, adrenal hypertrophy, Leydig cell hypertrophy with a NOAEL of 4mg/kg/day.

**Tier 3 Hazard assessment Output for EC1**
MoAs identified and adverse outcomes predicted:
Androgen receptor blocker - Male reproductive toxicity leading to infertility, hypospadias, delayed development. NOEL 25mg/kg. The NOEL effect level in multigeneration studies is predicted to be the same as for the in vivo assay based on reference t(as shown in Table ZZ) to others acting on same AOP as described by OECD ((http://www.oecd.org/chemicalsafety/testing/37478899.pdf ) . Using the criteria developed within CPL for assessing potency to set Specific Concentration Limits, EC1 would be of medium potency for reproductive toxicity and thus be in hazard category 2.

CYP induction leading to liver hypertrophy and possibly tumours. Anti-androgenicity leading to Leydig cell hyperplasia and possibly to tumours. Prudent to assume tumours with
CYP induction leading to liver hypertrophy and tumours, adrenal hypertrophy. Using the criteria developed within CPL for assessing potency to set Specific Concentration Limits, EC1 would be of medium potency for carcinogenicity and thus be in hazard category 2.

The nature of the effects seen in the 90 day study and the NOAEL of 4mg/kg would place EC1 in hazard category 1 for STOT RE.
Classification

- STOT RE Category 1
- Reproductive Toxicity Category 2*
- Carcinogenicity Category 2*

DNEL: 0.04mg/kg/day

| Chemical | MoA | Hershberger | Repro |
|---|---|---|---|

| | | | |
|---|---|---|---|
| Procymidone | antiandrogen | 10-30mg/kg | 25-50mg/kg |
| Finasteride | 5alpha reductase inhibitor | 0.2-1mg/kg | 0.1-1mg/kg |
| Linuron | antiandrogen | 30-100mg/kg | 25-50mg/kg |

Comparative potency of anti-androgens in Hershberger assay and multigeneration reproductive toxicity studies.

**Overall Exposure Tier 2 and Hazard Tier 3 Assessment**

The three uses are shown on the Exposure/Hazard Matrix. Hobby glue remains in a red segment where the options are to conclude as unsuitable for use or to move to higher tier assessments.

Use as an intermediate and in metal working fluid remain in an amber segment on the matrix which requires further consideration, including comparison of exposure and DNEL assessments. The DNEL derived from Tier 3 assessment is 0.04mg/kg. The exposures assessments from Tier 2 are 0.004mg/kg for use as a chemical intermediate and 0.002mg/kg for use in metal working fluid. There is enough margin of exposure and confidence in the hazard and exposure estimates to conclude that these uses would be safe provided the risk management measures assumed in the exposure assessment are in place.

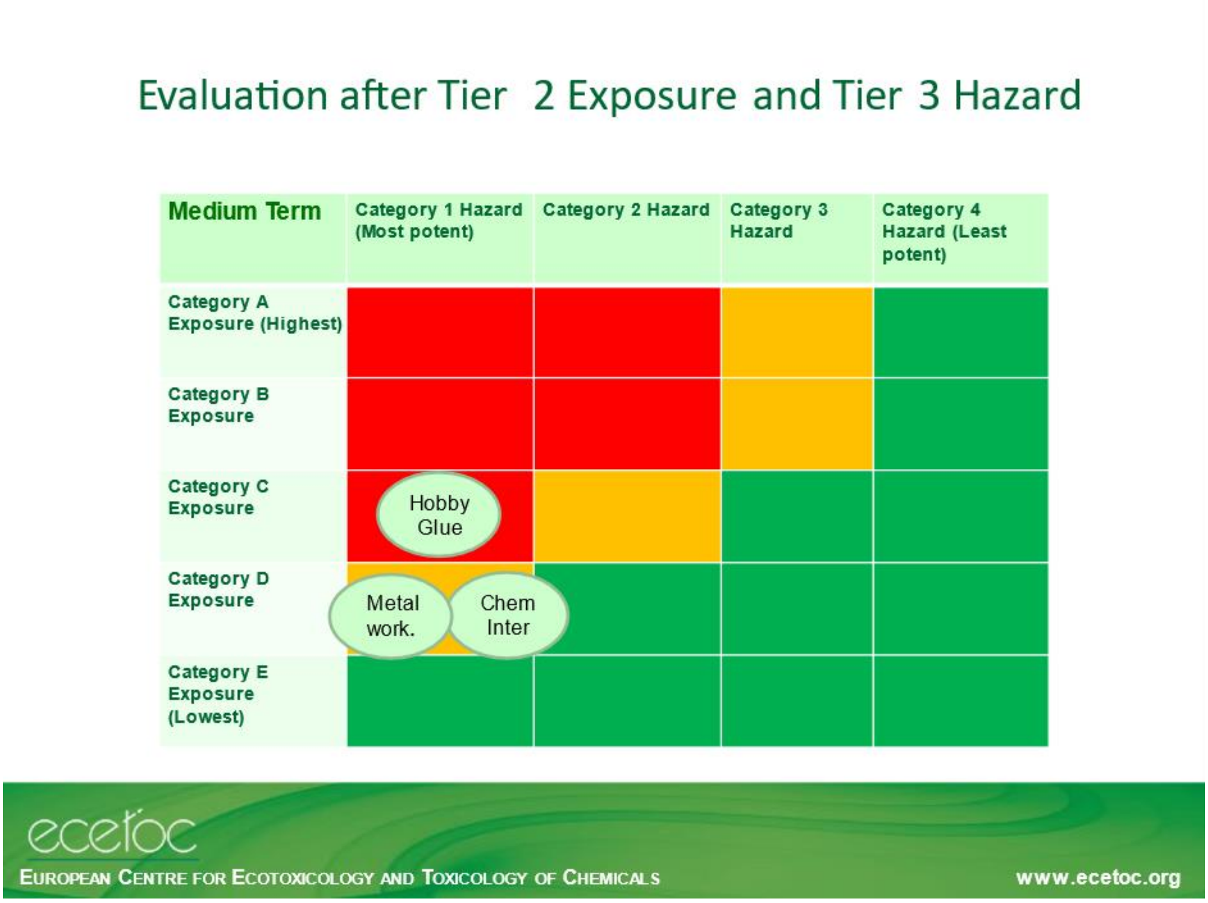

**Comparison with Conventional Assessment**
EC1 is vinclozolin which as a plant protection product has been the subject of a full conventional assessment. These are the relevant outcomes:

In the rat multigeneration reproductive toxicity study there were hypospadias and decreased ventral prostate weights at 50mg/kg and above. There was reduced sperm count at 100mg/kg. NOAEL 2.5mg/kg.

There were increased incidences of Leydig cell and adrenal tumours in the rat long term bioassay with a NOEL of 2.7mg/kg/day,

There were increased incidences of liver tumours in mouse long term bioassay with a NOEL of 24mg/kg/day.

Classification

- STOT RE Cat 1
- Repro Category 2*
- Carcinogenicity Category 2*

DNEL :0.024mg/kg

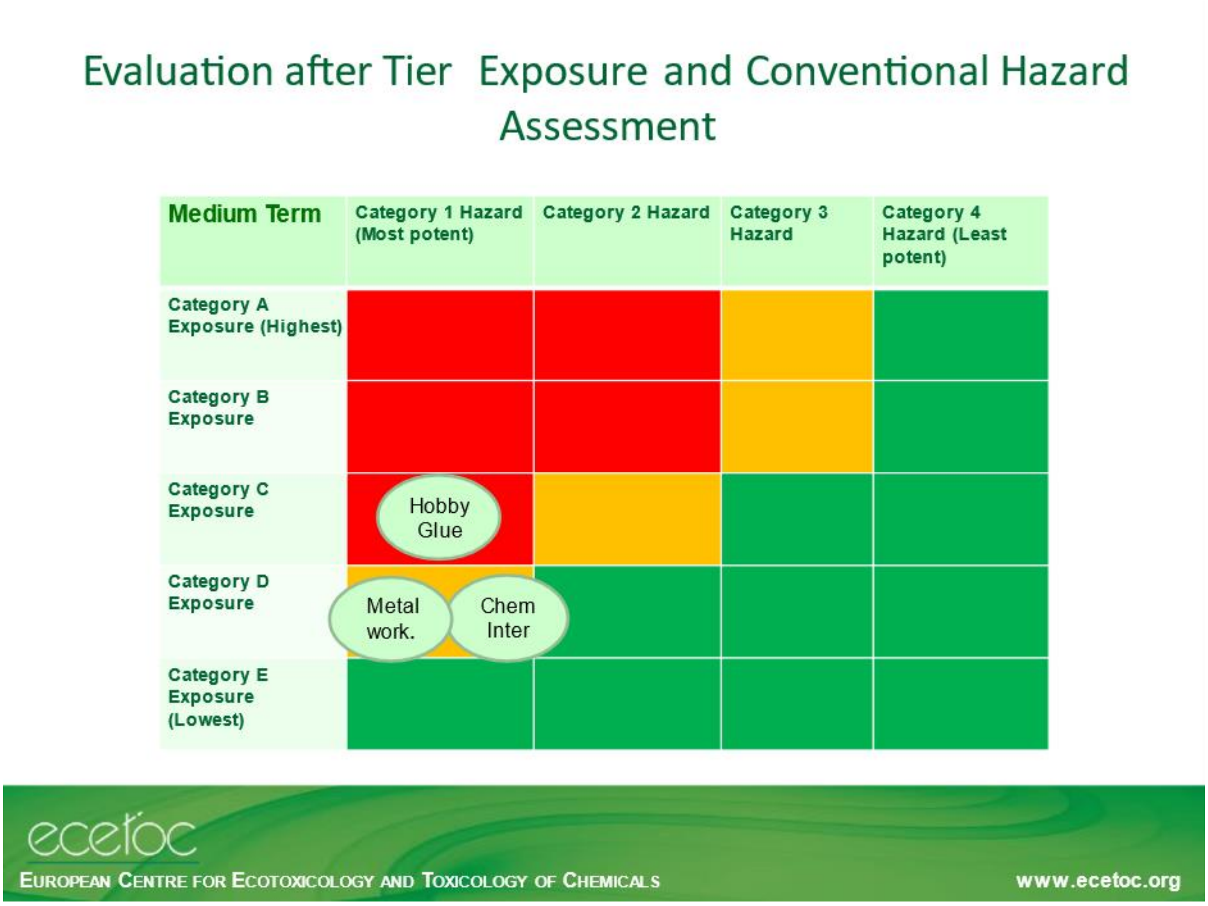


The three uses are shown on the Exposure/Hazard Matrix. The conventional assessment did not change the overall assessment.

**Comparison of Hazard Outputs**

The outputs from the Tiers can be compared. The main adverse outcomes were identified in Tier 2 based on in vitro data. The classification categories were changed to become less severe for carcinogenicity and reproductive toxicity. The DNEL increased by a factor of 10 moving from in vitro to in vivo but barely moved moving from targeted studies to full conventional studies.

- Tier 2 Output (0 animals used)
  - STOT RE Category 1
  - Reproductive Toxicity Category 1*
  - Carcinogenicity Category 1*
  - DNEL: 0.006mg/kg/day
- Tier 3 output (100 animals used)
  - STOT RE Category 1
  - Reproductive Toxicity Category 2*
  - Carcinogenicity Category 2*
  - DNEL: 0.04mg/kg/day
- Conventional Assay output (2200 animals used)
  - STOT RE Cat 1
  - Repro Category 2*
  - Carcinogenicity Category 2*
  - DNEL :0.024mg/kg

*Note on Classification for Carcinogenicity and Reproductive Toxicity
The current in CPL hazard categories for carcinogenicity and for reproductive toxicity are based on the strength of evidence for the presence of the hazard rather than on degree of hazard and therefore pose a problem. This makes it difficult to operate a scheme which incorporates degree of hazard (severity and potency) as part of the process. However, the degree of hazard is taken into account when assessing specific concentration limits (SCL) in preparations for substances which have been classified for carcinogenicity and reproductive toxicity. They are divided into high, medium and low potency bands based on results from the relevant studies – t25 for carcinogenicity and ED10 for reproductive toxicity. For this exemplification, chemicals predicted to show carcinogenicity have been assigned a category based on the SCL guidelines:

High potency – category 1
Medium potency – category 2
Low potency – category 3

**Example Chemical 2**

**Physicochemical data**

IUPAC name: chromen-2-one

Canonical SMILES: C1=CC=C2C(=C1)C=CC(=O)O2

Physicochemical data were obtained from PubCHem: Coumarin | C9H6O2 - PubChem (nih.gov)

**Tier 1 Usage and Exposure Assessment**

The uses of EC2 were listed and the relevant exposure scenarios from the ECETOC TRA ( https://www.ecetoc.org/tools/targeted-risk-assessment-tra/ ) were selected.

Uses requiring drum and small package filling (Liquid or solid in liquid). TRA PROC9

Uses requiring drum and small package filling (Aerosols/solids exposure). TRA PROC9

Use in Consumer Cleaning PC35:
- Dish washing products
- Laundry products
- All purpose cleaners
- Trigger Sprays

The relevant physicochemical data were entered into the TRA and the exposures estimated using worst case assumptions: possibility of aerosol, no modifying factors such as gloves, 100% absorption via inhalation and by dermal routes.

Each exposure was assigned a category as described in Annex x: Exposure Categorisation.

| Use | Inhalation mg/kg/day | Dermal mg/kg/day | Total dose per day | Duration | Exposure Category |
|---|---|---|---|---|---|
| Drumming in liquid | 0.55 | 6.9 | 10.7mg/kg | Intermittent | A |
| Drumming in solid | 3.7 | 7 | 7.8mg/kg | Intermittent | B – Check |
| Dish washing | 4.2 | 85 | 90mg/kg | Intermittent | A |
| Laundry | 4.2 | 85 | 90mg/kg | Intermittent | A |
| All purpose | 4.2 | 72 | 76mg/kg | Intermittent | A |
| Trigger spray | 56 | 29 | 85mg/kg | Intermittent | A |

**Hazard Assessment**
*Note: This hazard data for this evaluation are almost entirely extracted from:*
*Baltazar M, Cable S, Carmichael P, Cubberly R, Cull T, Delagrange M, Dent M, Hatherell S, Houghton J, Kukic P, Li H, Lee M-Y, Malcomber S, Middleton A, Moxon T, Nathanail A, Nicol B, Pendlington R, Reynolds G, Reynolds J, White A and Westmoreland C (2020) A Next-Generation Risk Assessment Case Study for Coumarin in Cosmetic Products Toxicol Sci 2020 Jul; 176(1): 236 - 252. doi: 10.1093/toxsci/kfaa048*

**Tier 0 Hazard Assessment**

All of the exposures are the highest category (Cat A) therefore the TTC does not apply.

**Tier 1 Hazard Assessment**

Tier 1 hazard assessment is based on in silico assessment.

EC2 is an aromatic organic chemical compound classified as a member of the benzopyrone family. The in silico tools ToxTree, OECD Toolbox, Derek Nexus, Meteor Nexus, TIMES, and molecular initiating events (MIE) ATLAS were run to predict the potential biological activity of coumarin, identify the active groups and predict its metabolic fate. ToxTree predicted that coumarin belongs to the Cramer class III and can bind to proteins and DNA via Michael addition and acyl transfer mechanism. Similarly, the OECD toolbox predicted binding to DNA and proteins via SN2 mechanisms after oxidation to epoxide. No positive results were obtained from the MIE ATLAS tool.

Based on the predicted rapid metabolism and the in silico predictions of toxic metabolite formation, Meteor Nexus was run to simulate the potential metabolic pathway of coumarin. Meteor Nexus identified hydroxylation as the main route of biotransformation followed by glucuronidation and sulfation with a total of 22 possible metabolites. Meteor Nexus also predicted the formation of epoxides (primary and secondary metabolites. Most primary, secondary, and tertiary metabolites were predicted to bind to proteins and DNA. Protein binding is a flag for skin sensitization potential; however, this endpoint was considered out of scope of this study. In summary, these in silico alerts indicated a need to investigate the genotoxicity potential of coumarin and its metabolites.

Tier 1 assessment indicated the potential for some adverse effects but could not provide an estimate of potency. Given that exposures are in Category A, it was necessary to move to Tier 2 Hazard Assessment.

**Tier 2 Hazard Assessment**

Tier 2 Hazard assessment is based on in vitro methodology. In vitro assessment is a three part process. Part 1 is determining what biological activity the chemical may have using a range of in vitro alerting assays. Part 2 is to use more specific assays to follow up on the activity indicated by part 1. Part 3 is to bring in assays and models based on kinetics and metabolism to provide an estimate of in vivo effect and no effect levels.

Part 1; Information on EC2 was gained from the range of assays in the **EPA ToxCast** program (https://www.epa.gov/chemical-research/exploring-toxcast-data-downloadable-data ).
EC2 was tested in 642 assays of the ToxCast panel, 18 of which were considered active.
The most credible hits in terms of quality of concentration-response and putative MoA were the 3 concordant positive hit-calls in the cell-free enzymatic assays for inhibition of monoamine oxidases (MAO) occurring at similar concentrations (15–19 µM).

The **genotoxicity** of EC2 was investigated with the ToxTracker assay. In this assay, a weak activation (between 1.5- and 2.0-fold) of the Rtkn-GFP marker, associated with DNA double-strand breaks, was observed in the presence of rat liver S9. However, no activation of the genotoxicity reporter Bscl2-GFP, which indicates DNA replication inhibition and induction of promutagenic DNA adducts, was observed in the absence or presence of S9. A weak activation was observed for the p53 response (Btg2-GFP reporter) in the presence of S9 which suggests general cellular stress. Similarly, exposure to EC2 without metabolic activation induced the oxidative stress reporter Srxn1-GFP, whilst in the presence of S9 both oxidative stress markers (Srxn1-GFP and Blvrb-GFP) were

activated. All controls caused GFP induction levels consistent with historical data and demonstrated the functionality of the mES reporter cell lines. No significant cytotoxicity occurred up to the maximum tested concentration of 1000 μM in the absence or presence of S9. In conclusion, the weak activation of DNA damage reporters in the presence of rat liver S9-mediated metabolism was not sufficient to classify EC2 as genotoxic in the ToxTracker assay. However, these results suggested that reactive coumarin metabolite(s) could induce DNA lesions secondary to oxidative stress, rather than directly interacting with DNA.

In vitro binding and enzymatic assays: Eurofins SafetyScreen44. The SafetyScreen44 panel includes 44 targets associated with in vivo adverse drug reactions. EC2 showed no significant effect in any of the targets.

Immunomodulatory screening assay: BioMap Diversity 8 Panel. To increase the biological coverage, a screening panel of 8 primary cell systems, that are stimulated to replicate complex cell and pathway interactions of vascular inflammation, immune activation, and tissue remodeling, was conducted. The most significantly affected biological readouts across all cell systems in the BioMap Diversity 8 Panel were associated with antiproliferative and tissue remodeling activities and the most sensitive cell system was the 3C endothelial cell system where antiproliferation (33%) was observed at the lowest dose tested (18.5 mM). No biomarkers were affected in the LPS or SAg-stimulated PBMC and endothelial cell coculture systems.

In vitro cell stress panel. Cellular stress-response assays are useful to characterize nonspecific biological activity which is not mediated via a specific protein/receptor interaction. In HepG2 cells, a dose-response with a CDS > 0.5 was only observed for 4 biomarkers (ATP, GSH, phospholipidosis, and IL-8) out of the 36 biomarkers across the 10 pathways with PoDs in the 500–800 μM range (Table 4). In NHEK cells, only the mitochondrial respiration parameters (oxygen consumption rate [OCR] and reserve capacity) decreased. The lowest PoD observed in the 2D models was 44 μM for reserve capacity at 1h, however the PoD increased to approximately 700 μM at the later timepoints.

High-throughput transcriptomics. Transcriptomics was applied as a broad nontargeted biological screen of in vitro cellular perturbation following coumarin treatment, to complement the targeted assays (eg, Eurofins SafetyScreen44, and BioMap). This study used 3 different cell lines (HepG2, MCF7, and 2D HepaRG) to extend biological coverage and address potential cellular variation in response to EC2 including any consequences of metabolism. In general, across the cell lines, treatment with coumarin resulted in limited gene expression changes at concentrations below 100 μM suggesting limited cellular effects at lower concentrations.

Pathway analysis and PoD determination. There were no clear indications of toxicity pathways from the in vitro studies, rather there were indications of mild general biological activity. A number of criteria were applied to determine Points of Departure which could be used for deriving DNELs. Including the mean of the 20 pathways with the lowest p value, or the 20 pathways with the lowest transcriptional BMDs and finally the lowest pathway BMD that meets the significant enrichment criteria. At the gene level this included both the mean BMD of 20 genes with largest fold change and the mean BMD of genes between 25th and 75th percentile. Only HepG2 met the recommendation that at least 20 pathways be detected to apply the pathway-level tests; no pathways were detected for MCF7, reflecting the DESeq2 results, and 17 pathways were detected using the 2D HepaRG cell model. Analysis of these changes using the Reactome pathway database corroborates a shift in metabolic responses for HepaRG 2D with pathways such as Metabolism, Biological oxidations, and

Phase 1-Functionalization of compounds amongst the lowest concentration related pathway responses. Where the number of pathways fell below 20, all relevant pathways were included in the analysis. Using this selection, the observed pathway-level PoDT ranged from 44 µM to 58µM across cell lines. Taking the lowest pathway identified as the PoDT this value reduces to between 31 and 38 µM .

In contrast, using the aggregated gene-level tests, the lowest PoDT was 1 µM derived using the MCF7 cell line. However, the low number of genes that pass the gene-level test filtering criteria, lack of detectable pathway responses, and results from the DESeq2 analysis indicate that evidence supporting this PoDT was weak. Using the more metabolically competent and physiological-relevant 2D HepaRG cell line resulted in a PODTs between 54 and 59 µM , where again the cytochrome P450 genes were amongst the most sensitive, similar to the pathway-level test results. The gene-level test PoDTs for HepG2s ranged between 6 and 17 µM and were slightly lower than the values derived for the pathway-level tests.

Metabolism in Primary Hepatocytes Using CYP2A6 Inhibition
EC2 metabolic stability screening in expressed human CYP and UGT isoforms (CYP1A2, CYP2A6, CYP2C8, CYP2C9, CYP2C19, CYP2D6, CYP3A4 and UGT1A1, UGT1A3, UGT1A6, UGT1A9, UGT2B7) identified CYP2A6 as the only enzyme metabolizing EC2. Pooled human cryopreserved primary hepatocytes in suspension at a cell density of 0.5 million cells per ml, were incubated at 37_C up to 90 min with 10 µM – 1mM EC2. Full scan liquid chromatography-mass spectrometry data were acquired using multienergy time-of-flight acquisition (Waters Xevo G2 Q-ToF in MSE mode).

In vitro to in vivo extrapolation (IVIVE) was predicted by a steady-state mass balance partitioning model based on published models parameterized to describe a specific in vitro assay setup and uses EC2's physicochemical properties.

**Summary from the Tier 2 Hazard assessment is:**
Non-genotoxic

MoAs identified and adverse outcomes predicted:
MAOI PoD – 12-40µM
CYP 450 pathways – possible liver enlargement – PoD 6-60µM
Carbonic anhydrase – PoD 21-62µM
Stress panel 500-800µM
No other pathways identified.
PoD range 6 - 60µM

IVIVE – In vivo PoD = 36 – 360mg/kg[1].

Footnote 1: IVIVE back calculated from Baltazar et al external dose to Cmax calculations.

**Output from Tier 2 Hazard**
Predict mild non-specific toxicity with hepatoenlargement. May lead to hepatocarcinogencity at high doses.
NOEL= 36-360mg/kg

In terms of **classification and DNEL**, EC2 would be:
Classification

– STOT RE No classification no significant toxicity
– Reproductive Toxicity No classification
– Carcinogenicity Possible Category 2

Long term and intermediate DNEL: 0.36 – 3.6mg/kg
Overall Category based on STOT RE NOEL = 2/3. Assume 2.

**Overall assessment after Tier 1 Exposure and Tier 2 Hazard Assessment**

The uses of EC2 are shown on the Exposure/Hazard Matrix. The consideration of exposure and of hazard shows category 2/3 hazard and category A exposure for both uses placing them in a red to amber segment. The options are to decide EC2 is not suitable for those uses or to refine exposure or hazard assessment. As exposure is Category A it was decided to refine exposure.

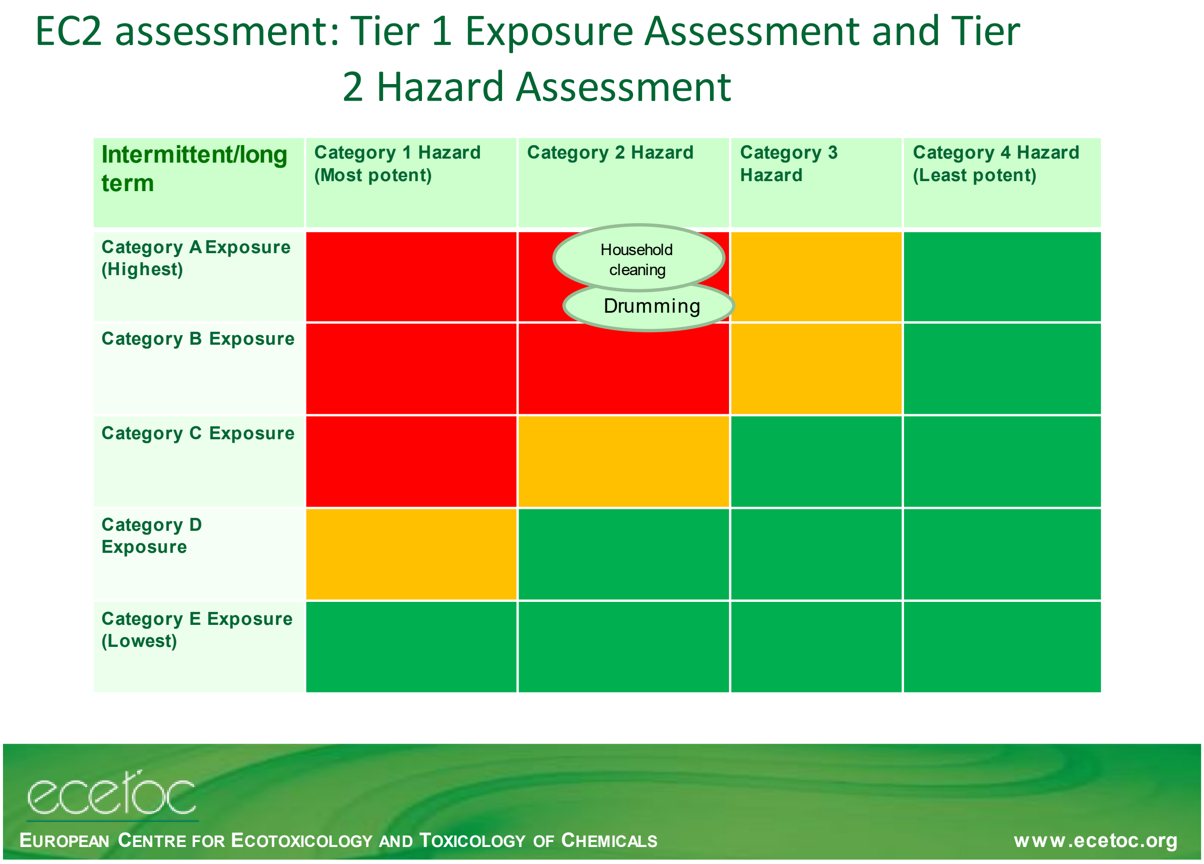


**Refine Tier 1 Exposure Assessment**

The first Tier 1 exposure assessment used worst case assumptions with no mitigation measures in place. Adjustments were made in the TRA calculations for reasonable precautions to be made for drumming and using specific consumer exposure determinants SCEDS to arrive at the exposure estimations shown below.

| Use | Inhalation mg/kg/day | Dermal mg/kg/day | Total dose per day | Duration | Exposure Category |
|---|---|---|---|---|---|
| Drumming in liquid | 0.05 | 1.4 | 1.45mg/kg | Intermittent | C |

| | | | | | |
|---|---|---|---|---|---|
| Drumming in solid | 0.37 | 0.14 | 0.5mg/kg | Intermittent | C |
| Dish washing | 0 | 85 | 85mg/kg | Intermittent | A |
| Laundry | 0.9 | 43 | 44mg/kg | Intermittent | A |
| All purpose | 0.85 | 14 | 15mg/kg | Intermittent | A |
| Trigger spray | 0.16 | 14 | 14mg/kg | Intermittent | A |

Table XX: Refined Tier 1 Exposure Assessment

Drumming in manufacture moves to Category C but household uses remain in Category A

**Overall Assessment after Tier 1 Refined Exposure and Tier 2 Hazard**

The uses are shown on the Exposure/Hazard Matrix. Drumming moved to an amber/green segment where it is prudent to compare exposure and DNEL. The exposure was calculated to be 0.5 to 1.45mg/kg per day and the DNEL was calculated to be 0.36-3.6mg/kg/day. Use in drumming is borderline acceptable. Household cleaning uses remain Category A placing the use in a red/amber segment, so it was decided to move to Tier 2 Exposure assessment.

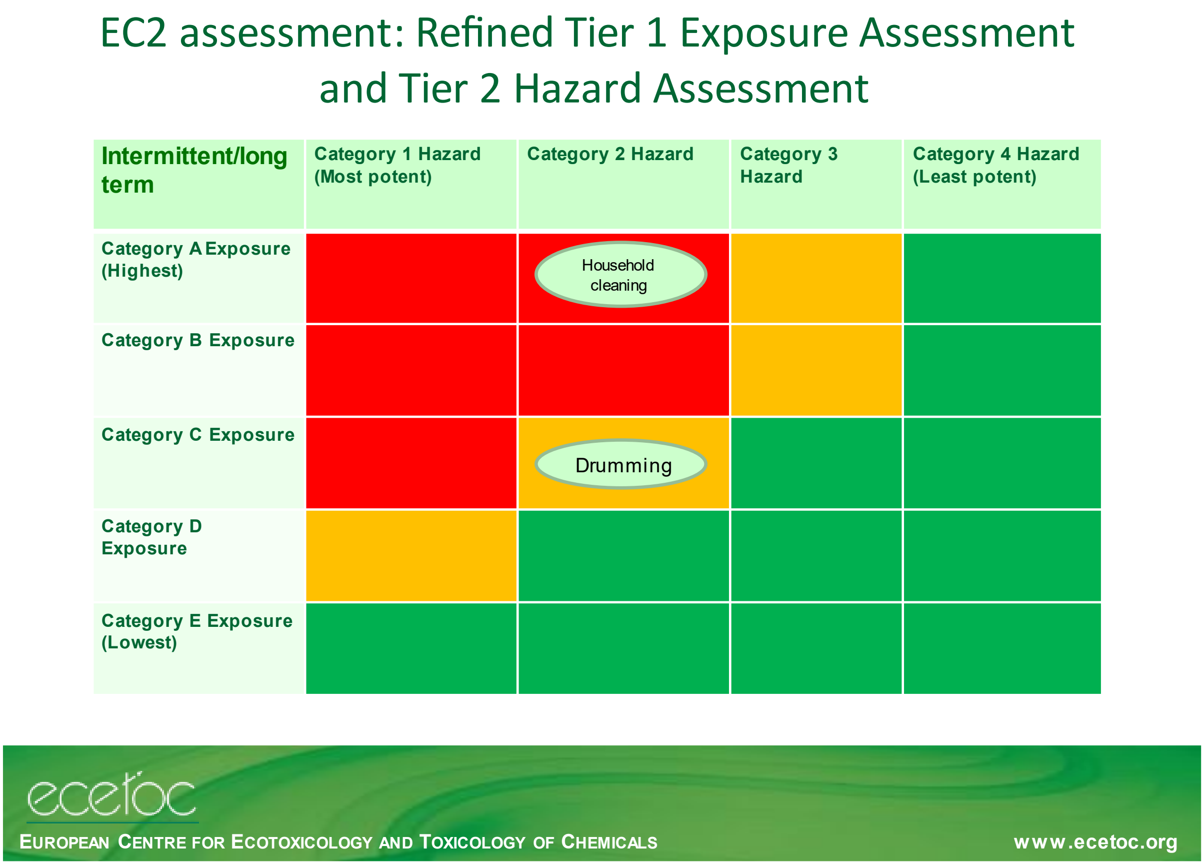


**Tier 2 Exposure Assessment**

Exposure for drumming was refined using the ART exposure model (Advanced REACH Tool - The Advanced Reach Tool - ART ).

Exposure for household use was refined using higher tier ConsExpo predicted exposure**.**

| Use | Inhalation mg/kg/day | Dermal mg/kg/day | Total dose per day | Duration | Exposure Category |
|---|---|---|---|---|---|
| Drumming in liquid | 0.03 | 2.1 | 2.1mg/kg | Intermittent | C |
| Drumming in solid | 0.007 | 0.5 | 0.5mg/kg | Intermittent | C |
| Dish washing | 0.18 | 0.36 | 0.5mg/kg | Intermittent | C |
| Laundry | 0.03 | 1.9 | 1.93mg/kg | Intermittent | C |
| All purpose | 0.8 | 2.4 | 3.2mg/kg | Intermittent | C |
| Trigger spray | 0.02 | 0.07 | 0.09mg/kg | Intermittent | C |

All uses were in category C.

**Overall Assessment for Tier 2 Exposure and Tier 2 Hazard**
The overall assessment for drumming as borderline acceptable did not change as a result of tier 2 exposure assessment. Household use was placed in an amber/green segment where it is prudent to compare exposure and DNEL. Exposures ranged from 0.09 to 3.2mg/kg/day and the DNEL was calculated to be between 0.36 and 3.6mg/kg/day, also indicating borderline acceptable use.

# EC2 assessment: Tier 2 Exposure Assessment and Tier 2 Hazard Assessment

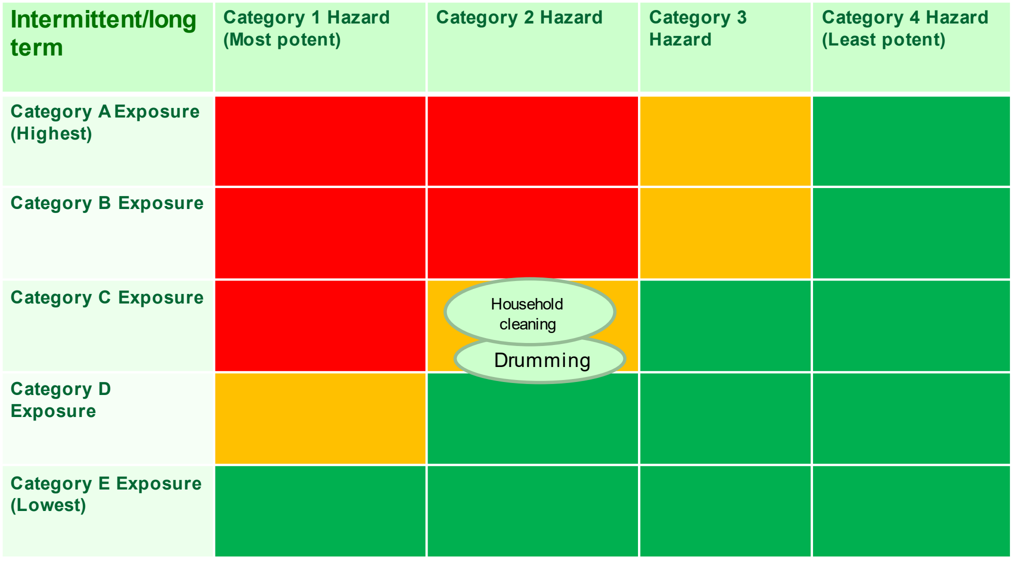


EUROPEAN CENTRE FOR ECOTOXICOLOGY AND TOXICOLOGY OF CHEMICALS www.ecetoc.org

**Comparison with Conventional Assessment**
EC2 is coumarin which has been the subject of conventional toxicology studies.  A similar

Carlton et al. (1996) administered Sprague–Dawley rats a diet containing 0, 333, 1000, 2000, 3000, or 5000 ppm coumarin for two years (equivalent to intakes of 0, 13, 42, 87, 130 or 234 mg/kg/day in males and 0, 16, 50, 107, 156 or 283 mg/kg/day in females). Animals in the lower dose groups c.were also exposed in utero and throughout lactation. An increased incidence of cholangiofibrosis, cholangiocarcinoma, and parenchymal cell tumors were seen in males and females in the highest dose group only and an increase in parenchymal cell tumors in male rats were observed at 3000 ppm. The authors considered these dose groups to have exceeded the MTD (maximum tolerated dose) based on dramatic
dose-related decreases in body weight gain. No increase in tumors was seen in dose groups up to 2000 ppm. In comparison, the NOEL for effects on liver weight and morphology was 1000 ppm for males (42 mg/kg/day) and 333 ppm for females (16 mg/kg/day).

Carlton, B. D., Aubrun, J. C., & Simon, G. S. (1996). Effects of coumarin following perinatal and chronic exposure in Sprague-Dawley rats and CD-1 mice. *Fundamental and Applied Toxicology*, *30*(1), 145–151. https://doi.org/10.1006/faat.1996.0051

**Output from Conventional Animal Studies**
Mild non-specific toxicity with hepatoenlargement.  Hepatocarcinogencity at high doses.

NOEL= 16-42mg/kg

In terms of **classification and DNEL**, EC2 would be:
Classification

- STOT RE No classification no significant toxicity
- Reproductive Toxicity No classification
- Carcinogenicity Category 2

Long term and intermediate DNEL: 0.16-0.42mg/kg
Overall Category based on STOT RE NOEL = 2

**Overall Assessment for Tier 2 Exposure and Conventional Hazard**
Both uses were placed in an amber segment where it is prudent to compare exposure and DNEL.
Drumming exposure was 0.5 to 2.1mg/kg/day with DNEL calculated to be 0.16-0.42mg/kg/day, borderline acceptable.
Household use exposures ranged from 0.09 to 3.2mg/kg/day and the DNEL was calculated to be 0.16-0.42mg/kg/day, also indicating borderline acceptable use.

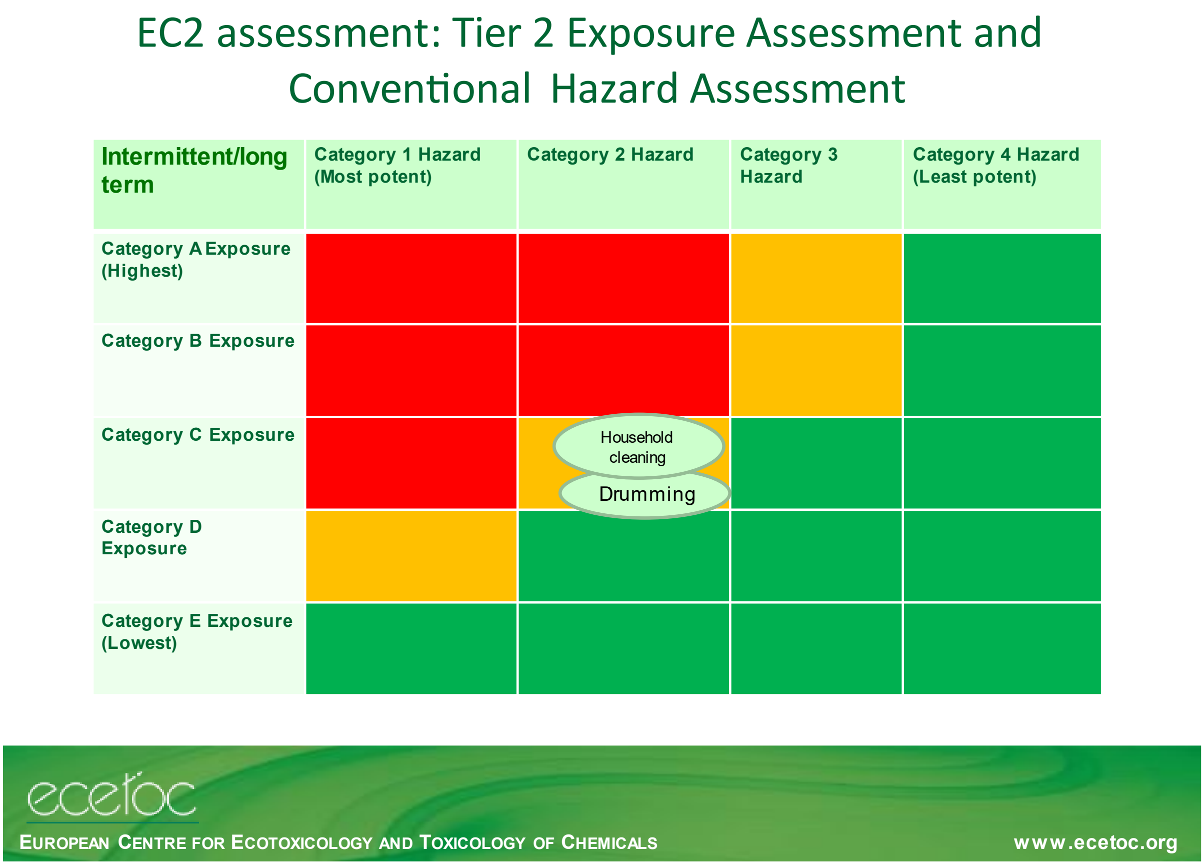


**Comparison of Hazard Outputs**
The outputs from Tier 2 based on vitro data and from the conventional assessments are similar in terms of the nature of the effects, the classification categories and DNELs.

**Output from Tier 2 Hazard (0 animals used)**

Predict mild non-specific toxicity with hepatoenlargement.  May lead to hepatocarcinogencity at high doses.
NOEL= 36-360mg/kg

In terms of **classification and DNEL**, EC2 would be:
Classification
- STOT RE No classification no significant toxicity
- Reproductive Toxicity No classification
- Carcinogenicity Possible Category 2

Long term and intermediate DNEL: 0.36 – 3.6mg/kg
Overall Category based on STOT RE NOEL = 2/3.  Assume 2.

**Output from Conventional Animal Studies (c. 1000 animals used)**
Mild non-specific toxicity with hepatoenlargement.  Hepatocarcinogencity at high doses.
NOEL= 16-42mg/kg

In terms of **classification and DNEL**, EC2 would be:
Classification
- STOT RE No classification no significant toxicity
- Reproductive Toxicity No classification
- Carcinogenicity Category 2

Long term and intermediate DNEL: 0.16-0.42mg/kg
Overall Category based on STOT RE NOEL = 2

**Example Chemical 3**

A third chemical was evaluated for hazard but no exposure assessment was made.

**Physicochemical data**

IUPAC Name: 2-phenoxyethanol

Canonical SMILES: C1=CC=C(C=C1)OCCO

Physicochemical data were obtained from PubCHem : [2-Phenoxyethanol | C8H10O2 - PubChem (nih.gov)]

Tier 0 – As a hazard evaluation was done, the use of the TTC was inappropriate.

Tier 1 – In silico assessment.
Tier 1 starts with an assessment based on the structure about what is known about molecules with similar structures and uses a series of tools
EC3 is a preservative which can inhibit malate dehydrogenase in bacteria, which is part of the citric acid cycle and therefore conserved across most, if not all, species including humans. Sequence homology was used to determine the level of sequence identity and similarity between the bacterial and human enzymes. To further understand whether the human isoforms of the bacterial target enzyme could be a target for phenoxyethanol the docking of phenoxyethanol inside the active site of the relevant enzymes in the presence of NAD+/NADH was simulated using CDOCKER module in Discovery Studio 2018 (Gagnon, Law and Brooks, 2016). Re-docking of endogenous substrates (malate and oxaloacetate) in the same active site was used as a positive control.
*In silico* tools that were used to identify potential MoA of EC3 were: OECD QSAR Toolbox v. 4.1 (https://www.oecd.org/chemicalsafety/risk-assessment/oecd-qsar-toolbox.htm), Derek Nexus v 5.0.2 Lhasa Ltd.), COSMOS nuclear Receptors Binding profilers (https://knimewebportal.cosmostox.eu/com.knime.enterprise.server/#login /), MIE (Molecular initiating event) Atlas (Allen *et al.*, 2018), models of the Collaborative Estrogen Receptor Activity Prediction Project (CERAPP) (Mansouri *et al.*, 2016) and the Collaborative Modeling Project of Androgen Receptor Activity (CoMPARA) (Grisoni, Consonni and Ballabio, 2019). Only alerts relevant to systemic toxicity (including genetic toxicity) were considered.
The results of the in silico assessments are summarised:

- Derek Nexus: inactive (negative) in the Ames assay
- OECD Toolbox: *in vivo* mutagenicity (micronucleus) in rodents: alert for H-acceptor-path3-H-acceptor
- MIE Atlas: no alerts
- CERAPP and CoMPARA: no binding predicted
- COSMOS profilers: potential binding to Thyroid Hormone Receptor (THR).

In silico tools were also used to predict the likely metabolism of EC3. The major routes of metabolism predicted by the software (Meteor Nexus v. 3.1.0 v, Lhasa Ltd.) are summarised in **Error! Reference source not found.** and comprised: aromatic hydroxylation, oxidative dealkylation, glucuronidation, oxidation of primary alcohols and O-sulphonation. Meteor Nexus predicted 19 unique metabolites (30 in total). Eight of these metabolites were predicted to be formed directly from the parent compound (level 1 metabolism).

The major metabolite was predicted to be phenoxy acetic acid (PAA) which was also assessed using the in silico tools. The results for PAA were the same as for phenoxyethanol with the following additional alerts:

- Derek Nexus: alert for plausible hepatotoxicity,
- OECD Toolbox: binding to the oestrogen receptor (ER), binding to proteins, hepatoxicity.
- COSMOS profilers: potential binding to both ER and THR.

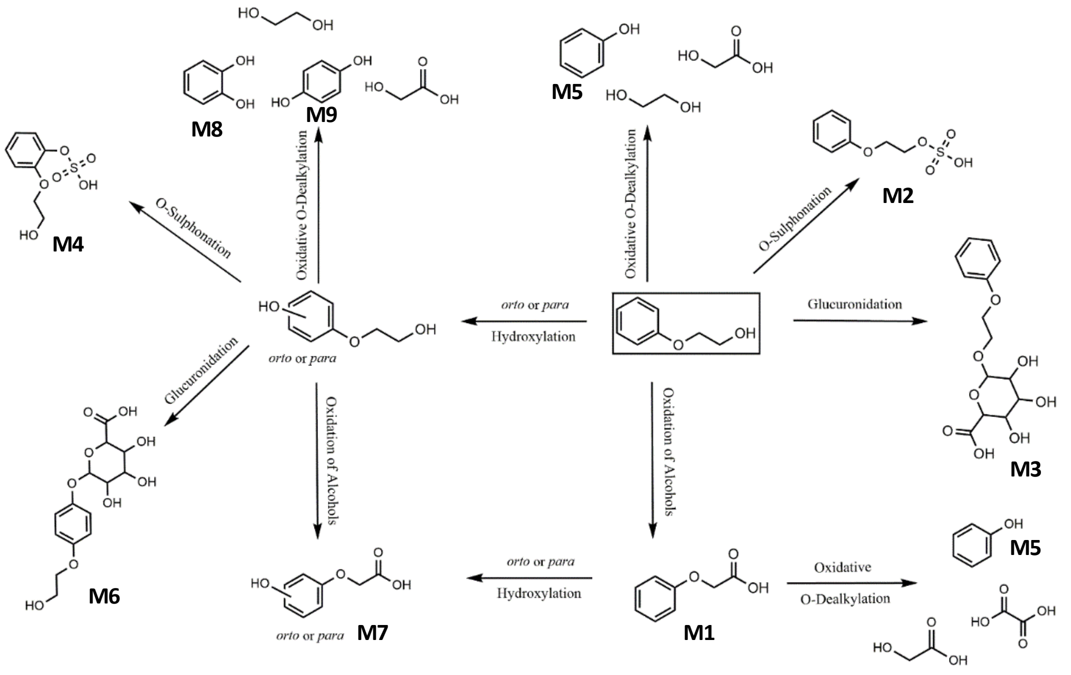


Tier 2: In vitro assessments

A range of in vitro assays were performed as shown in table x

| Assay | Cell types | Rationale for generation |
|---|---|---|
| SafetyScreen44™ pharmacological profiling | N/A (initial profiling is cell-free, with positive responses followed-up *in vitro*) | Panel of 44 targets recommended by 4 major pharmaceutical companies as significant liabilities in drug development (Bowes *et al.*, 2012) |
| Cell stress panel* | HepG2 | Cellular stress underlies many adverse health effects and is likely to be especially relevant for cosmetic ingredients with a low affinity for specific biological targets. |
| Transcriptomics (TempO-Seq) (dose response) | MCF7, HepG2, HepaRG | Transcriptional activity across the entire genome provides wide biological coverage; adverse cellular changes will result in changes to gene transcription |

The SafetyScreen44™ assays showed no significant effect for phenoxyethanol in any of the binding and enzymatic assays at the 10 µM concentration. The highest reported inhibition was 12.1% and was attributed to variability of the signal around the control level. The results showing the percentage inhibition of the binding of a radioactively labelled ligand and percentage inhibition of control enzyme activity are shown in Table 6.

In a panel of biomarkers to identify PoDs in HepG2 cells for risk assessment based on several key cellular stress pathways, mitochondrial function and general cellular health, phenoxyethanol did not

show any activity at concentrations up to 1000 µM (Hatherell *et al.*, 2020). This panel, consisting mostly of high content imaging assays, assessed several biomarkers of cellular respiration including cellular ATP levels, oxygen consumption rate, and mitochondrial reserve capacity) showed that even at a high concentration phenoxyethanol did not cause any effects. This contrasted with substances known to cause cellular stress that were shown to perturb these pathways (Figure 10), and therefore alleviated concern that phenoxyethanol could cause adverse effects by interfering with human MDH.

*Table 7 NOTEL values for phenoxyethanol across 3 cell lines calculated using BMD modelling.*

| **Gene Tests** | **HepaRG** | **MCF-7** | **HepG2** |
|---|---|---|---|
| **$BMD_{10}$ of pathway with the lowest $BMD_{10}$ (µM)** | 552.90 | 760.33 | 232.00 |
| **$BMDL_{10}$** | 220.92 | 512.84 | 171.25 |
| **$BMDU_{10}$** | 911.72 | 1648.51 | 557.20 |

In addition a wide range of assays (ToxCast) were assess EC3, most ToxCast hits were disregarded because the dose-response or variability in the data were not suggestive of a true effect of treatment. The only hit that was not disregarded on this basis was an increase in serum amyloid A1 in vascular smooth muscle cells in assay BSK_CASM3C_SAA_up, which gave an $AC_{50}$ value of 16.6 µM. This hit was from one assay in the BioMap set of assays, which consists of 8 different cell types chosen to identify and characterize perturbations relevant to human inflammatory disease (Houck *et al.*, 2009). Because this was a single hit on one protein target with no clearly mechanistically related hits in other cell types or inflammatory markers,

*In vitro* metabolism identification in PHH showed the major metabolites of EC3 to be phenoxyacetic acid. The PBK sub-model for the internal exposure of PAA predicted a 95$^{th}$ percentile kidney $C_{max}$ of 69 µM and $AUC_{24}$ of 1569 µM. This indicated the importance of including a NGRA for PAA at levels predicted in kidney tissue.

PAA had been shown to be formed in both HepG2 and HepaRG cell It was therefore possible to interpolate the $C_{max}$ and $AUC_{24}$ at the NOTEL for EC3 in both these cell types (Figure 11). This showed that at the NOTEL for HepG2 and HepaRG cells the cellular $C_{max}$ of PAA was approximately 217 µM and 492 µM respectively, and the $AUC_{24}$ was approximately 3550 µM/L and 7163 µM/L respectively. Note this was only done in the cell lines that showed appreciable formation of PAA (HepG2 and HepaRG). A similar approach was used to calculate the $AUC_{24}$ value for EC3 in the media at the relevant PoD.

Given the lack of compelling evidence that EC3would be active in humans via a specific mechanism, it was considered to fit into the 'no defined biological target or pathway' category as described in the US EPAs blueprint for computational toxicology (Thomas *et al.*, 2019), which provides a hypothesis that can be explored further in Tier 2. The aim of Tier 2 was therefore to build confidence in the exposure and metabolism predictions and to use higher tier tools to ensure broad enough biological coverage of data regarding phenoxyethanol. The latter considered whether MDH and SENP8 were expressed in the cell lines used to help understand if these could be targets of phenoxyethanol, since *in vitro* confirmation that phenoxyethanol can inhibit these enzymes in eukaryotic cells may indicate a more specific toxicity (AOP) that needs to be examined.

Phenoxyethanol showed limited biological activity across all the assays. The only ToxCast hit that showed a credible dose-response was related to SAA1, although this biomarker was only raised at the highest concentration tested, and no pathways relevant to an acute inflammatory response were affected in any of the transcriptomics assays or biomarkers relevant to inflammatory pathways in the cell stress panel. This single hit was therefore considered to be of no biological relevance.

Neither the SafetyScreen44™ nor the cellular stress panel provided sufficient evidence of phenoxyethanol being active to identify a PoD. The PoD from the transcriptomics assessments (the NOTELs) therefore provided important information for the risk assessment.
Phenoxyethanol showed a very low level of transcriptional activity in MCF7, HepaRG and HepG2 cells at 24-hours. The $BMDL_{10}$ of 171 µM in HepG2 cells was taken as the most sensitive PoD for the risk assessment. This PoD is highly conservative because in this cell line only 1 affected pathway (signal transduction) was identified.

- A PBK was developed to characterise the internal exposure to phenoxyethanol. The model was verified using human urinary data
- *In vitro* metabolite identification in PHH was carried out to confirm/refute in silico predictions. These data were used to develop a sub-PBK model so that internal exposure to the major metabolite can also be characterised
- Additional *in vitro* biokinetic refinements included measuring the concentration of phenoxyethanol and its major metabolite in cells and media under the same conditions as the bioactivity assays described below. This allows a quantitative *in vitro* to *in vivo* extrapolation to be performed for both the parent and the major metabolite.

| **Chemical** | **Scenario** | **PoD** |
|---|---|---|
| | | **$C_{max}$ µM** |
| **Phenoxyethanol** | Worst case (BMDL/P95 Exposure) | 171 |
| **Phenoxyethanol** | Mean (BMD/Mean Exposure) | 232 |
| **Phenoxyethanol** | Best case (BMDU/P05 Exposure) | 557 |
| **PAA** | Worst case (BMDL/P95 Exposure) | 217 |
| **PAA** | Mean (BMD/Mean Exposure) | 249 |
| **PAA** | Best case (BMDU/P05 Exposure) | 359 |

Dosing EC3 gives a ration of 1:10 of parent to metabolite PAA. As the in vitro PODs are similar for parent and for PAA, the reverse dosimetry calculation to derive the in vivo PoD is based on PAA. 1µm PAA is equivalent to a dose of 0.0087mg/kg EC3 and the PoD range of 217-359µM PAA is equivalent to an in vivo PoD of 1.9-3.1mg/kg. An uncertainty factor of 100 would give a DNEL of 0.019-0.031mg/kg.
No clear mode of action has emerged and EC3 should be considered to show general toxicity which is likely to be seen in the liver and/or kidneys.
**Summary from the Tier 2 Hazard assessment is:**
Non-genotoxic

MoAs identified and adverse outcomes predicted:
No pathways identified.
PoD range EC3 171-557µM PAA(major metabolite) 217-359 µM

IVIVE – In vivo PoD = 1.9-3.1mg/kg[1].
Footnote 1: IVIVE back calculated from Bent et al external dose to Cmax calculations.

**Output from Tier 2 Hazard**

Predict non-specific toxicity, possibly liver toxicity.
NOEL= 1.9-3.1mg/kgmg/kg

In terms of **classification and DNEL**, EC3 would be:
Classification

- STOT RE No classification no significant toxicity
- Reproductive Toxicity No classification
- Carcinogenicity Possible No classification

Long term and intermediate DNEL: 0.019-0.031mg/kg
Overall Category based on STOT RE NOEL = 1

Safety review of phenoxyethanol when used as a preservative in cosmetics - Dréno - 2019 - Journal of the European Academy of Dermatology and Venereology - Wiley Online Library

2-phenoxyethanol - Registration Dossier - ECHA (europa.eu)

90 day rat study:
Drinking water

| | Male | Female |
|---|---|---|
| 1250mg/l | 96mg/kg | 163mg/kg |
| 2500mg/l | 185mg/kg | 313mg/kg |
| 5000mg/l | 369mg/kg | 652mg/kg |
| 10000mg/l | 687mg/kg | 1000mg/kg |
| 20000mg/l | 1514mg/kg | 1702mg/kg |

CLINICAL SIGNS AND MORTALITY
Soiled fur around genitalia was observed in all animals administered with 20,000 mg/L test substance and in a few animals exposed to 5000 and 10,000 mg/L. One male rat in the 20000 mg/L dose group did not survive the 13th study week, all other animals survived until necropsy.

BODY WEIGHT AND WEIGHT GAIN
After 13 weeks a significant decrease in body weight was observed in both sexes of the 20,000 mg/L dose group (±19% lower compared to the control group). Decrease in body weight was also observed in females administered with 10,000 mg/L test substance (±8% lower compared to the control group)

FOOD CONSUMPTION AND WATER CONSUMPTION
During the entire study a decrease in food consumption was observed in males and females in the 20,000 mg/L dose groups (±20% relative to controls) and in females in the 10,000 mg/L dose group (±10% relative to controls). During the entire study water consumption was decreased in females administered with 20,000 mg/L. The decrease in females was approximately 30-40%. In males dosed with 5000, 10000, and 20000 mg/L decreased water consumption was observed untill the 9th week of the study.

HAEMATOLOGY

Red blood cell count and platelet count were reduced in the 10000 and 20000 mg/L dose groups of both sexes. In males platelet count was also reduced in animals exposed to 5000 mg/L. The reduction of platelets in the 5000 mg/L dose group was less then 10% compared to controls and no changes were observed in coagulation parameters. Therefore no evidence of a functional effect was present. In the 10000 and 20000 mg/L dose groups in females and in the 20000 mg/L dose group in males haemoglobin was reduced. MCV and MCH were increased in males (10000 and 20000 mg/L) and females (20000 mg/L). In females administered with 20000 mg/L an increased reticulocyte count was measured.

CLINICAL CHEMISTRY
In males exposed to 20000 mg/L test substance a reduction in total protein, glucose, sodium, calcium and an increase in A/G ratio, total cholesterol, phospholipids, urea nitrogen, potassium was observed. In the 10000 mg/L dose group only the effects on total protein, cholesterol, phospholipids, sodium and potassium were present. No effects were observed at lower dose levels. In female exposed to 20000 mg/L increases in A/G ratio, ALP, and urea nitrogen was observed. In the 10000 mg/L dose group total protein was decreased and urea nitrogen was increased.

URINALYSIS
Statisticaly significant decrease in pH was observed after administration of 20000 mg/L in both sexes. This could be the result of excretion of 2-phenoxyacetic acid, the major metabolite of the test substance.

ORGAN WEIGHTS
In the 10000 mg/L dose groups, the relative liver weight was increased in males and females. In addition in females absolute adrenal weight was decreased and relative kidney and brain weights were increased. In males of the 20000 mg/L dose group a decrease in absolute thymus, testes, heart, lung, and spleen was observed. In addition an increase in relative testes, lung, kidney, liver, brain, and thyroid was determined. In females of the 20000 mg/L dose group decreases in absolute thymus, adrenals, ovaries, heart, lungs, and spleen weights were determined. Relative kidney, liver, and brain weights were increased.

HISTOPATHOLOGY
In 2 males and 1 female dosed with 10000 mg/L, slight urothelial hyperplasia of the renal pelvis was observed. Slight to moderate urothelial hyperplasia was observed in 6 males of the highest dose group. In 2 and 7 females dosed with 10000 and 20000 mg/L test substance, respectively slight to moderate urinary bladder transitional epithelial hyperplasia was observed. Slight urinary bladder transitional epithelial hyperplasia was also observed in one male of the high dose group.
NOAEL: 5000mg/l male 369mg/kg female 652mg/kg

Output from Conventional Studies:
**Summary from the Tier 2 Hazard assessment is:**
Non-genotoxic
No specific toxicity. Liver weight increased, kidney and bladder effects. Red blood decreases.
NOAEL: 5000mg/l male 369mg/kg female 652mg/kg

**Output from Tier 2 Hazard**
Non-specific toxicity, possibly liver toxicity.
NOEL= 300 -600/kgmg/kg

In terms of **classification and DNEL**, EC3 would be:
Classification

- STOT RE No classification no significant toxicity
- Reproductive Toxicity No classification
- Carcinogenicity Possible No classification

Long term and intermediate DNEL: 3-6mg/kg
Overall Category based on STOT RE NOEL = 2
